# Studies of the antitumor mechanism of action of dermaseptin B2, a multifunctional cationic antimicrobial peptide, reveal a partial implication of cell surface glycosaminoglycans

**DOI:** 10.1371/journal.pone.0182926

**Published:** 2017-08-10

**Authors:** Célia Dos Santos, Sabah Hamadat, Karen Le Saux, Clara Newton, Meriem Mazouni, Loussiné Zargarian, Mickael Miro-Padovani, Patricia Zadigue, Jean Delbé, Yamina Hamma-Kourbali, Mohamed Amiche

**Affiliations:** 1 Laboratoire (CRRET), EAC 7149 CNRS, University Paris Est Créteil, Créteil, France; 2 University Paris Est Créteil, Créteil, France; 3 Mondor Institute of Biomedical Research, INSERM U955 Team 7, School of Medicine, University Paris Est Créteil, Créteil, France; 4 BPA, CNRS UMR 8113 Bâtiment IDA, Ecole Normale Supérieure de Cachan, Cachan, France; Duke University School of Medicine, UNITED STATES

## Abstract

Dermaseptin-B2 (DRS-B2) is a multifunctional cationic antimicrobial peptide (CAP) isolated from frog skin secretion. We previously reported that DRS-B2 possesses anticancer and antiangiogenic activities *in vitro* and *in vivo*. In the present study, we evaluated the antiproliferative activity of DRS-B2 on numerous tumor cell lines, its cell internalization and studies of its molecular partners as well as their influences on its structure. Confocal microscopy using ([Alexa594]-(Cys^0^)-DRS-B2) shows that in sensitive human tumor cells (PC3), DRS-B2 seems to accumulate rapidly at the cytoplasmic membranes and enters the cytoplasm and the nucleus, while in less sensitive tumor cells (U87MG), DRS-B2 is found packed in vesicles at the cell membrane. Furthermore FACS analysis shows that PC3 cells viability decreases after DRS-B2 treatment while U87 MG seems to be unaffected. However, "pull down" experiments performed with total protein pools from PC3 or U87MG cells and the comparison between the antiproliferative effect of DRS-B2 and its synthetic analog containing all D-amino acids suggest the absence of a stereo-selective protein receptor. Pretreatment of PC3 cells with sodium chlorate, decreases the antiproliferative activity of DRS-B2. This activity is partially restored after addition of exogenous chondroitin sulfate C (CS-C). Moreover, we demonstrate that at nanomolar concentrations CS-C potentiates the antiproliferative effect of DRS-B2. These results highlight the partial implication of glycosaminoglycans in the mechanism of antiproliferative action of DRS-B2. Structural analysis of DRS-B2 by circular dichroism in the presence of increasing concentration of CS-C shows that DRS-B2 adopts an α-helical structure. Finally, structure-activity-relationship studies suggest a key role of the W residue in position 3 of the DRS-B2 sequence for its antiproliferative activity.

## Introduction

Cancer is one of the leading causes of morbidity and mortality worldwide. Nevertheless, in the last decades numerous advances have been made in cancer therapy, even though present treatments do not cure more than 50% of cancer cases. Unfortunately, the conventional chemotherapeutic agents that target cancer cells are often associated with adverse side effects observed in healthy fast-dividing cells. Furthermore, these agents can depress the immune system and cancer cells frequently develop resistance to many anti-cancer drugs [1,2]. Therefore, to evade drug resistance and reduce cytotoxicity affecting healthy cells, the development of alternative/innovative compounds with new mechanisms of action and increased selectivity for cancer cells is crucial [3].

In this context and to improve or find new therapeutic molecules against cancer, peptide-like or peptidomimetic drugs derived from either the exploration of biodiversity or from chemical synthesis became one of the new research targets. A group of interesting peptides from natural sources are antimicrobial peptides (AMPs). Indeed, in recent years, an increasing number of articles show that these AMPs are in fact multi-functional peptides such as anti-cancer agents, immuno-modulators, chemokines, vaccine adjuvants, or regulators of innate defense [4].

Based on the spectrum of the anticancer activity of these AMPs, the cationic antimicrobial peptides (CAPs) can be divided into two broad classes [5]: the first includes peptides that are highly effective against bacteria and cancer cells, yet not against healthy mammalian cells; the second class includes peptides that are toxic to bacteria, cancer and non-cancerous cells. However, some AMPs possess no anticancer activity. This raises the question about the molecular mechanisms and structural characteristics of CAPs involved in the toxicity of cancer cells.

Our team explores an original model, the skin of an Amazonian frog of the genus *Phyllomedusa bicolor* that has proven in recent years to be a huge reservoir of bioactive peptides. The CAPs dermaseptin B2 and B3 isolated from *P*. *bicolor*, are members of the DRS-B family, they have high membrane-lytic antibacterial activity whereby they quickly kill Gram-positive and Gram-negative bacteria, yeast, protozoa and filamentous fungi, and have little or no hemolytic activity [6–9]. DRS-B2 also known as adenoregulin [10] is an α-helical amphipathic polycationic polypeptide with a molecular mass of 3180 Da (_NH2_-GLWSKIKEVGKEAAKAAAKAAGKAALGAVSEAV-_NH2_). DRS-B2 is the most abundant member and the most active peptide of the DRS-B family with a minimal inhibitory concentration in the micromolar range [6, 7, 11].

Recently, we reported significant antitumor activity of DRS-B2 and DRS-B3 [12]. In addition, we showed that DRS-B2 inhibits the proliferation and colony formation of human prostate and mammary tumor cells, and the proliferation and capillary formation of endothelial cells *in vitro* [13]. Furthermore, DRS-B2 inhibited tumor growth of the xenografted human prostate adenocarcinoma cell line PC3 *in vivo*. Analyses of the mechanism of action of DRS-B2 on tumor PC3 cells demonstrated a rapid increase in the amount of cytosolic lactate dehydrogenase and no changes in mitochondrial membrane potential. Confocal microscopy analyses revealed that DRS-B2 could interact with the tumor cell surface, aggregate and penetrate into the cells. Despite these studies, the mechanism of action of DRS-B2 remains unclear and incomplete.

In the present study, we investigated the antitumor activities of DRS-B2 on a wide range of human tumor cells *in vitro*. On one hand, using the most sensitive cell line (PC3 prostate cell line) and the least sensitive cell line (U87MG glioblastoma cell line) to DRS-B2 treatment, we investigated the molecular and cellular mechanisms whereby DRS-B2 is able to impair tumor cell proliferation. On the other hand, we compared the effect and the mechanism of DRS-B2 analogs to identify potential cellular targets of DRS-B2 for prostate cancer therapy.

## Materials and methods

### Materials

Dulbecco’s modified Eagle’s medium (DMEM) GlutaMAX containing 4.5 mg/L D-glucose and sodium pyruvate, RPMI-1640 and α-MEM GlutaMAX, fetal bovine serum (FBS), phosphate buffered saline (PBS), and gentamycin [50 mg/mL] were provided by Invitrogen Life Technologies Corporation (Cergy Pontoise, France). Bovine serum albumin (BSA) and agar were from Sigma-Aldrich (St Quentin en Fallavier, France), and trypsin (0.05% trypsin and 0.02% EDTA in Dulbecco’s PBS (D-PBS)) was from the PAA Laboratories (Les Mureaux, France). All the human tumoral cell lines tested were obtained from ATCC (Manassas, VA, USA). Crystal violet staining was from Gurr-Searle Diagnostic (High Wycombe, Bucks, England). Purified mouse anti-human EMMPRIN/CD147 antibodies was from BD Pharmingen (Bedford, UK)

### Synthesis and purification of DRS-B2 and its analogs

DRS-B2 and its analog containing amino acids in the D-configuration ([all.D]-DRS-B2) and DRS-B2 in which the W residue at position 3 was substituted by the F residue ([W/F]^3^-DRS-B2) were synthesized using solid-phase 9-fluorenylmethoxycarbonyl (Fmoc) chemistry procedures on an automated microwave-assisted peptide synthesizer (liberty 1, CEM). The sequences of all the peptides are shown in Table 1. All Fmoc-protected amino acids and Rink amide 4-Methylbenzhydrylamine resin (100–200 mesh, 0.38 mmol/g) were purchased from Iris. Each peptidyl-resin and side chain protection bonds were cleaved by incubation in a mixture of 95% trifluoroacetic acid, 2.5% triisopropylsilane, and 2.5% water for 2 h at room temperature [14]. The resulting mixture was filtered to remove the resin, and the crude peptides were precipitated with cold terbutylmethylether (TBME). They were recovered by centrifugation at 5000 *g* for 15 min at 4°C, washed three times with cold TBME, dried, dissolved in 10% acetic acid, and lyophilized. The crude peptides were purified by reverse-phase high-performance liquid chromatography using a C18 column, and the homogeneity and identity of the peptides were assessed by MALDI-TOF mass spectrometry (Voyager DE PRO, Applied Biosystems) and analytical RP-HPLC.

**Table 1:** Names and amino acid sequences of DRS-B2 analogs.

Name	Amino acid sequence
DRS-B2	GLWSKIKEVGKEAAKAAAKAAGKAALGAVSEAV-_NH2_
[all.D]-DRS-B2	glwskikevgkeaakaaakaagkaalgavseav-_NH2_
[W/F]^3^-DRS-B2	GL**F**SKIKEVGKEAAKAAAKAAGKAALGAVSEAV-_NH2_
Biotin-(GGG)-DRS-B2	**Biotin-GGG**GLWSKIKEVGKEAAKAAAKAAGKAALGAVSEAV-_NH2_
[Alex594]-Cys^0^-DRS-B2	**Alexa594-C**GLWSKIKEVGKEAAKAAAKAAGKAALGAVSEAV-_NH2_

The L-amino acids are in capital letters and the D-amino acids are in small letters.

### Synthesis of biotin-[GGG]-DRS-B2

DRS-B2 containing 3 additional glycine (G) residues at its amino terminal extremity ([GGG]-DRS-B2) used as spacer between the peptide and biotin was synthesized as described above. The biotin (10 equivalents of the peptidyl-resin) was then activated with an Hexafluorophosphate de 2-(1H-benzotriazol-1-yl)-1,1,3,3-tetraméthylaminium / N,N Diisopropylethylamine (HBTU/DIEA) solution in N-methyl-2-pyrrolidone (NMP) and coupled manually to the peptidyl resin. Between each step, several NMP washes were carried out to remove traces of piperidine, or TFA and excess unreacted amino acid. The biotin-peptidyl-resin and side chain protection bonds were cleaved and purified as described above.

### Chemical synthesis of [Alexa594]- (Cys^0^)-DRS-B2

We first synthesized DRS-B2 with an additional cysteine residue (Cys^0^)-DRS-B2 at its amino terminus by solid phase peptide synthesis according to the Fmoc strategy as described above. The spectrometry analysis of (Cys^0^)-DRS-B2 after purification by RP-HPLC shows that the monoisotopic masses are identical between the measured (M+H^+^ = 3282.79 g/mol) and the calculated mass (MH^+^ = 3281.83 g/mol) (S1 and S2 Figs). The Alexa594 fluorophore was then grafted through its maleimide function on the SH group of (Cys^0^)-DRS-B2. Purification of the complex [Alexa594]-(Cys^0^)-DRS-B2 was carried out by exclusion chromatography on a Sephadex G10 column and eluted with 10% acetic acid. The absorbance of each fraction was determined at 280 nm and 625 nm based on the absorbance of the W residue and the fluorescence emission of the Alexa excited at 594 nm, respectively (S3 Fig). Fractions 4 to 10 in S3 Fig corresponding to maximum peak absorbance at 280 nm and an emission maximum at 625 nm after excitation at 594 nm were pooled and lyophilized and the concentration determined using nanodrop^®^.

### Cell culture and *in vitro* proliferation assay

The tumor cell lines adherent of the prostatic adenocarcinoma PC3, DU145 and LnCap were grown in RPMI-1640 medium supplemented with 5% (v/v) for PC3 and DU145, and with 10% (v/v) FBS for LnCap and 50 μg/ml gentamycin (complete medium). The human U87MG glioblastoma cell line was routinely maintained in α-minimum essential medium containing 10% (v/v) FBS. All cell cultures were maintained at 37°C and 7% CO_2_ in humidified atmosphere.

For proliferation assay, the cells were seeded at a density of 10^4^ cells/well in 24-well plates (1.91 cm^2^) in 0.5 mL complete medium and incubated at 37°C in a controlled humidified 7% CO_2_ environment. On the first and third days after plating, the cells were treated with DRS-B2 at different concentrations. Twenty four hours after the last treatment, adherent cells were washed with PBS1X, fixed with absolute ethanol, and cell counting was carried out with crystal violet staining (Gurr-Searle Diagnostic; High Wycombe; Bucks, England), as previously described [13].

### Test of sodium chlorate on PC3 cell proliferation

PC3 cells were seeded at a density of 10^4^ cells/well in 24-well plates (1.9 cm^2^) in 0.5 mL complete medium and incubated at 37°C in a controlled humidified environment with 7% CO_2_. On the first, third, and fifth days after plating, the cells were treated with DRS-B2 at different concentrations. Twenty-four hours after the last treatment, adherent cells were washed with PBS 1x, fixed with absolute ethanol, and cell count was carried out with crystal violet staining as previously described [13]. When tested in the presence of sodium chlorate, PC3 cells were first seeded in a 24-well plate as described above and on the second day of incubation, increasing concentrations of sodium chlorate (0 to 80 mM) were added and crystal violet staining was performed on the fourth day.

### Anti-proliferative activity of DRS-B2 on PC3 cells in the absence or presence of sodium chlorate

PC3 cells were seeded in 24-well plates with 10^4^ cells/well. Sodium chlorate (10 mM) was added on the second day of incubation, and the following day DRS-B2 (2.5, 5 or 10 μM) was added. After 4 hours, cell count was performed using the crystal violet technique.

### Anti-proliferative activity of DRS-B2 on PC3 cells in the absence or presence of CS-C

The peptides at 3 different concentrations (2.5, 5 or 10 μM) were pre-incubated or not with increasing concentration of CS-C (0–3.3 nM) at 37°C for 15 min and then added to the cells on the second and the fourth day of incubation. The cell count was performed with the crystal violet technique on the fifth day of cell incubation.

### Lactate dehydrogenase (LDH)-release assay

The LDH release assay was performed as previously described [13]. Briefly, PC3 cells were grown in a 96 well plate (1.500 cells/well/100 μL) in complete medium and treated with DRS-B2 (2.5 μM) with or without sodium chlorate (10 mM) and various CS-C concentrations (0–3.3 nM). Cell membrane integrity was evaluated by measuring the LDH activity released into the culture media 3 hours after DRS-B2 exposure. The CytoTox96 non-radioactive cytotoxicity assay (Promega; Charbonnières-les-Bains, France) was performed according to the manufacturer’s instructions and quantified by measuring the absorbance at 490 nm. The 100% cytotoxicity corresponds to the LDH released with the DRS-B2 treatment alone at 2.5 μM.

### Treatment of PC3 and U87MG cells by [Alexa594]-(Cys^0^)-DRS-B2 and immunofluorescent staining

PC3 or U87MG cells were seeded onto 6-well plates containing glass coverslips at a concentration of 300 000 cells per well in complete culture medium. After 48 hours, the cells were treated with DRS-B2 coupled to Alexa (Alexa-(Cys^0^)-DRS-B2) at 2.5 μM for either 5 min or one hour. The cells were washed with 1X PBS and then fixed with 4% paraformaldehyde (PFA) for 10 min at room temperature. They were then washed three times with 1X PBS. The saturation of non-specific sites was carried out with PBS 1X containing 3% BSA for 1 hour at room temperature. After saturation, the cells were washed three times in 1X PBS and incubated overnight in a humid chamber at 4°C with a primary antibodies (anti-CD147 antibody / EMMPRIN). The cells were washed three times with 1X PBS then incubated with the appropriate secondary antibodies coupled to a fluorochrome that was diluted in PBS/1% BSA. After fixation, the membrane was visualized by anti-CD147 antibodies labeled with an anti-mouse antibody A488 (green) and nuclei by DAPI (blue). The confocal images were acquired using an Olympus IX81 inverted microscope (60x oil immersion objective NA 1.25) with a spinning disk confocal system DSU (Olympus, Rungis, France), coupled to a CCD camera R2 Orca (Hamamatsu Corporation, Japan). Image processing was done using the Image J software and the program Zoom in Images and Stack.

#### Cell viability with FITC-Annexin-V / 7-Amino-Actinomycin D (7-AAD) double staining

Cells were grown in a 6 wells plate and treated or not with DRS-B2 as described previously [13]. One hour after exposure, cell viability was evaluated by flow cytometry analysis with FITC-Annexin-V (A-V) and 7-Amino-Actinomycin D (7-AAD) staining. Medium and trypsinized cells were collected and washed with PBS. After centrifugation cells were suspended in PBS to obtain a cell density of 0.5x10^6^ cells per mL. One mL of this cell extract was centrifuged, suspended in 200 μL PBS, transferred to a microtiterplate with round bottom and centrifuged again. Resulted cell pellet was resuspended in 200 μL of Binding Buffer 1x (BD Pharmingen^™^), containing 5 μL of FITC-Annexin-V (BD Pharmingen^™^) and incubated during 10 minutes in dark at room temperature. The cells were washed with PBS and incubated with 200 μL of Binding Buffer 1x containing 7-AAD (final concentration 1 μg/mL) (BD Pharmingen^™^)) during 5 minutes in dark at room temperature. Flow cytometry analysis was performed with Cyan ADP LX7 analyzer (Beckmann Coulter)

### Cell-based ELISA binding assay

PC3 cells were seeded in triplicate on 96-well plates at 2.10^4^ cells/well, incubated overnight, and subsequently starved for 24 h. Before the binding experiment, the wells were incubated with 300 μl/well RPMI containing 3% BSA (w/v), for 1 h at room temperature. Binding of biotinylated DRS-B2 was achieved in RPMI containing 1% (w/v) BSA for 2 h at room temperature. Unbound biotinylated peptide was removed by washing the cells three times with phosphate buffered saline (PBS) containing 1% (w/v) BSA (washing buffer), and fixing them with PBS supplemented with 4% (w/v) paraformaldehyde (PFA) for 10 min at room temperature (100 μl/well). The cells were washed three times with washing buffer, and non-specific binding sites were blocked for 1 h at room temperature with PBS containing 3% (w/v) BSA (300 μl/well). The bound peptide was characterized using a peroxidase-labeled antibody to an anti-biotin antibody diluted 1:2000 in PBS containing 0.5% BSA (w/v), for 1 h at room temperature. Peroxidase activity was detected using 3,3,5,5-tetramethylbenzidine dihydrochloride according to the supplier's instructions. Absorbance was measured at 450 nm. For competition experiments, PC3 cells were incubated with 1 μM biotinylated DRS-B2 and increasing concentrations of DRS-B2. The characterization of the bound peptide was performed as above.

### DRS-B2 “pull down” experiments

Cells were lysed in RIPA buffer supplemented with protease inhibitors (Sigma Aldrich). Samples (1.5 mg) were first pre-cleared with Streptavidin-Sepharose beads (Amersham) for 30 min at 4°C to avoid non-specific interactions. They were then incubated for 3 h at 4°C with 20 μl (10% w/v) N-Biotin-DRS-B2/Streptavidin-Sepharose coupled beads representing 5μM N-Biotin-DRS-B2. The beads were then washed extensively with lysis buffer and once with buffer containing 4 mM Hepes pH 7.4, and 10 mM NaCl. The bound protein was eluted by incubating the beads for 10 min at room temperature in 100 mM glycine pH 2.8 and the eluates were neutralized with 1 M Tris pH 8. The eluates were diluted 4 times and the beads were suspended in 2 times concentrated electrophoresis sample buffer containing SDS, heated and analyzed by SDS-polyacrylamide gel electrophoresis (PAGE). The gels were stained with silver staining from Sigma.

### Circular dichroism (CD)

CD measurements were performed in a Jobin Yvon CD6 dichrograph linked to a PC microprocessor as described [15]. Briefly, measurements were calibrated with (+)-10-camphorsulfonic acid and performed with 10 mM DRS-B2 diluted in water alone or with increasing concentrations of chondroitin sulfate C (1–20 μM) at 25°C using a quartz cuvette (Hellma) with a path length of 0.1 cm. Spectra, recorded in 1 nm steps, were averaged over five scans and corrected for the base line. The CD spectra were deconvoluted using CDNN Software [16] Circular dichroism measurements are reported as Δε/n, where Δε is the dichroic increment (M^-1^. cm^-1^) and n is the number of residues in the peptide. The α-helix content of peptides was obtained using the relation: Pα = -[Δε222nm x10] (Pα: percentage of α-helix; Δε222nm: CD per residue at 222 nm) [17].

### Fluorescence

Emission spectra were recorded on a Jobin-Yvon Fluoromax II instrument (HORIBA Jobin-Yvon, France) equipped with an Ozone-free 150 W xenon lamp. The excitation wavelength was 280 nm and the emission spectra were acquired at 300–360 nm. At least five measurements for each titration point were recorded with an integration time of 1 s. The DRS-B2 concentration in water was 2 μM, and the CS-C concentration varied from 0 to 10 μM. Tryptophan fluorescence was determined by subtracting spectra without peptide.

### Statistical analysis

The statistical analyses were performed using the GraphPad PrismTM version 4.00 software from GraphPad Software Inc. (San Diego, CA, USA). The results are expressed as the means ± standard deviation (SD) or standard error mean (SEM) of at least three determinations for each test from three independent experiments. Statistical analyses were carried out using the unpaired t-test. The statistical significance of the differences is given as * p < 0.05; ** p < 0.01; *** p < 0.001; ns: not significant.

## Results

### Effect of DRS-B2 on the *in vitro* proliferation of solid tumor cells

The *in vitro* antiproliferative effect of DRS-B2 was tested on human tumor cell lines of different origins such as mammary carcinomas (MDA-MB 231, MDA-MB 453, BT-474), androgen-dependent or independent prostatic adenocarcinomas (PC-3, DU145, LnCaP), glioblastomas (U87MG, U138MG, U373MG), pancreatic carcinomas (PANC-1, BxPC-3, MiaPaCa2) and melanomas (A375, SK-Mel-28, HT-144). No effect of DRS-B2 was observed on the proliferation of human fibroblasts used as control cells (13). As shown in S4 Fig, DRS-B2 presented a dose-dependent antiproliferative effect on different cell lines. Comparative studies indicated that prostatic cell lines seemed to be the most sensitive to DRS-B2 with a concentration that inhibits 50% of cell growth (GI_50_) between 0.71 to 2.65 μM (Table 2). In contrast, within the range of the concentrations tested, DRS-B2 had no effect on the proliferation of the glioblastoma and mammary carcinoma cell lines. Therefore, we decided to use PC3 (as sensitive) and U87MG (as resistant) cell lines to study the mechanism of action of DRS-B2 on cancer cells. PC3 cells were used as representative of the GI50 mean of the three prostate cell lines treated.

**Table 2:** Concentration of DRS-B2 that inhibits 50% cell growth (GI_50_) extracted from S2 Fig. Proliferation of each cell type was performed in plastic 24-well plates (1.91 cm^2^; cell density of 1x10^4^ cells/well/0.5 mL). On the first and third day after plating, the cells were treated with DRS-B2 at different concentrations ranging from 0 to 10 μM. Twenty four hours after the last treatment cell counting was performed with crystal violet staining as described in Materials and Methods section. Results are represented in peptide concentration inhibiting 50% of the cell growth GI50 ± SD of at least three determinations.

Tumor cells	GI_50_ (μM)
U-87MG	>10
U-138MG	>10
U-373MG	>10
MDA MB231	>10
BT-474	>10
MDA MB 453	>10
Panc-1	>10
BxPC3	2.31 ± 0.41
MIACaPA2	7.91 ± 1.40
HT-144	5.08 ± 0.72
SK-Mel-28	>10
A375	>10
DU145	0.71 ± 0.55
PC3	2.17 ± 0.48
LNCaP	2.65 ± 1.09

### Cell internalization mechanism of DRS-B2

To study the internalization of DRS-B2, we choice PC3 and U87MG cells as sensitive and resistant cell models, respectively. Our previous studies on cell internalization of DRS-B2 in PC3 cells, using anti-DRS-B2 polyclonal antibodies, revealed that this peptide accumulates at the plasma membrane and then seems to penetrate inside the cell by a mechanism that remains to be elucidated [13]. To further explore this mechanism, we synthesized an analog of DRS-B2 containing a fluorophore Alexa-594 grafted onto its N-terminal amino acid ([Alexa594]-(Cys^0^)-DRS-B2) (S1, S2 and S3 Figs). This compound was synthesized in view of minimizing the auto-fluorescence and the non-specific interactions of the anti-DRS-B2 antibodies observed in our previous studies. We used [Alexa594]-(Cys^0^)-DRS-B2 to study its localization by confocal microscopy in the two human tumor cell lines mentioned above, PC3 and U87MG.

Previously we examined DRS-B2 at the surface of PC3 cells by confocal microscopy after 1 hour of treatment [13]. To determine if the difference in sensitivity to DRS-B2 between PC3 and U87MG cells could be due to the localization of the peptide at the cell surface, we treated the PC3 and U87MG cell lines for 5 min and 1 hour with 2.5 μM of [Alexa594]-(Cys^0^)-DRS-B2. The distribution of the fluorescence was then analyzed by confocal microscopy and the transmembrane glycoprotein CD147 was used as plasma membrane marker as previously described [13].

**Fig 1:**
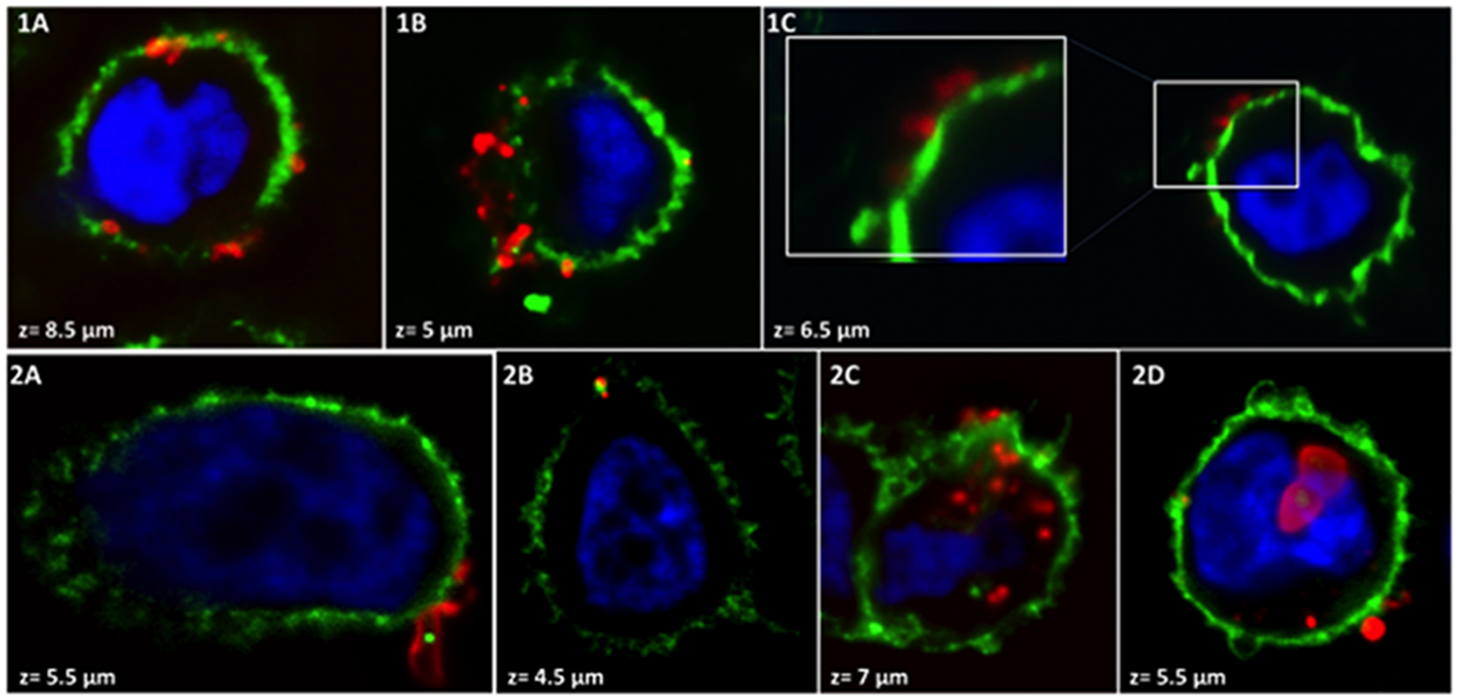
Immunostaining of the membrane of PC3 cells after treatment with [Alexa594]—(Cys^0^) -DRS-B2. PC3 cells were treated with 2.5 μM [Alexa594]—(Cys^0^) -DRS-B2 (red) for either 5 min. (1A-C) or 1 hour (2A-D). After fixation, the membrane was visualized by the anti-CD147 antibody labeled with an anti-mouse antibody A488 (green) and nuclei by DAPI (blue). The confocal images were obtained using an Olympus IX81 inverted microscope (60x oil immersion objective NA 1.25) with a spinning disk confocal system DSU (Olympus), coupled to a CCD camera R2 Orca (Hamamatsu Corporation, Japan). Image processing was performed using the ImageJ software, and the program Zoom in Images and Stack. z is the distance from the base to the top of the cell in 0.5 μm steps. The boxes are zoom lenses, the zoom factor = 2.

**Fig 2:**
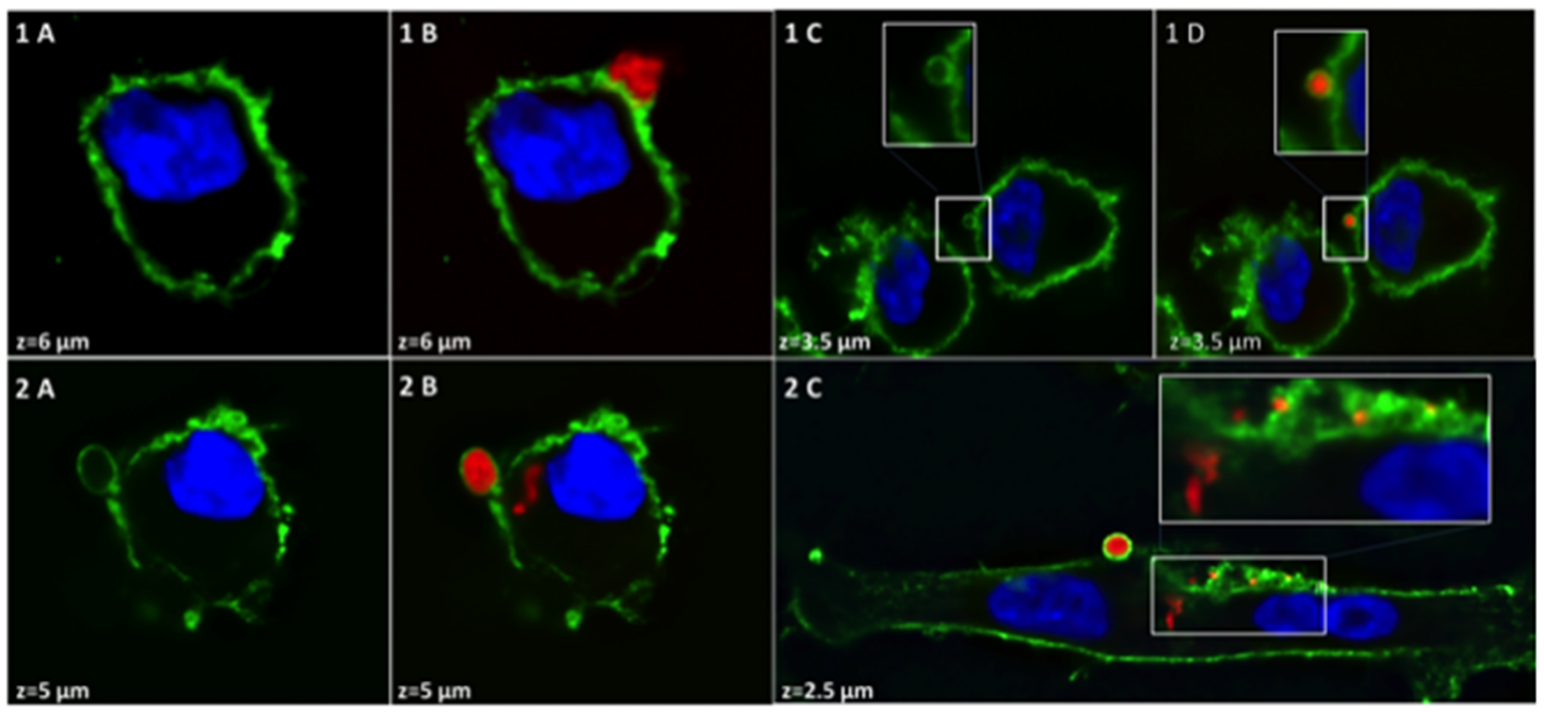
Immunostaining of the membrane of U87MG cells after treatment by [Alexa594]—(Cys^0^) -DRS-B2. U87MG cells were treated with 2.5 μM of [Alexa594]—(Cys^0^) -DRS-B2 (red) for either 5 min. (1A-D) or 1 hour (2A-C). After fixation, the membrane was visualized using anti CD147 antibody labeled with an anti-mouse antibody A488 (green) and nuclei by DAPI (blue). The confocal images were acquired using Olympus IX81 inverted microscope (60x oil immersion objective NA 1.25) with a spinning disk confocal system DSU (Olympus), coupled to a CCD camera R2 Orca (Hamamatsu Corporation, Japan). Image processing was performed using the program Zoom in Images and Stack. z is the distance from the base to the top of the cell in 0.5 μm steps. The boxes are zoom lenses, the zoom factor = 2.

Figs 1 and 2 represent particular localizations and different states of DRS-B2 aggregation on PC3 or U87MG cells respectively with a global maximum projection of the RGB (Red, Green and Blue) composite stacks. Incubation of [Alexa594]-(Cys^0^)-DRS-B2 on PC3 cells for 5 min highlighted the presence of a few molecules of DRS-B2 at the extracellular and at the transmembrane domains (Fig 1, panels 1A-1C). The same observation was made after 1 hour of treatment (Fig 1, panels 2A-2D) but in some cases, the DRS-B2 particles appeared as highly condensed structures at the extracellular membrane (Fig 1, panel 2A). As observed previously by Zoggel *et al*. [13], [Alexa594]-(Cys^0^)-DRS-B2 was observed in the cytoplasm (Fig 1, panel 2C) and in the nucleus (Fig 1, panel 2D).

Fig 2, panels 1A-1D and panels 2A-2C present the immunolocalization of [Alexa594]-(Cys^0^)-DRS-B2 on the membrane of U87MG cells after 5 min and after 1 hour of treatment respectively. An extracellular aggregate of DRS-B2 was observed (Fig 2, panel 1B) and appears to be partially wrapped by membrane extensions of the cell as shown in Fig 1, panel 1D. After one hour of treatment, the DRS-B2 aggregates were enveloped by the cell membrane of the U87MG cells (Fig 2, panels 2B and 2C). Furthermore, DRS-B2 was found in the cytoplasm but there was no evidence that the intracellular particles were surrounded by membrane structures.

**Fig 3:**
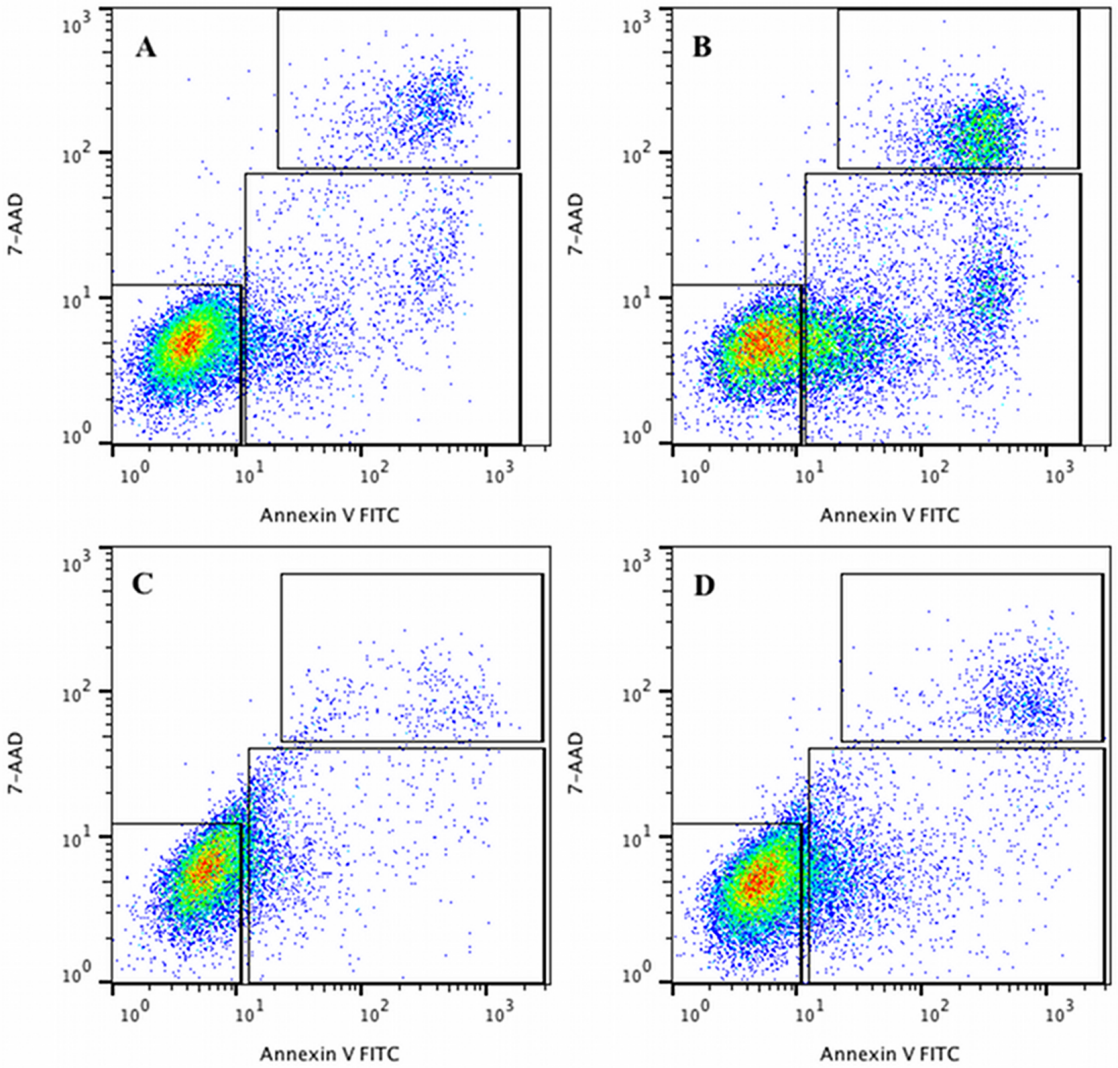
PC3 and U87MG cell viability after DRS-B2 treatment. PC3 and U87MG cells were treated with DRS-B2 (2.5 μM) for one hour. Then cells where double stained with FITC-Annexin-V (A-V) and 7-AAD and analyzed by flow cytometry. The cell viability was observed by measuring the amount of A-V and 7-AAD negative and positive cells. Counter diagrams show an example of A-V/7-AAD double staining of PC3 cell control (A), PC3 treated with DRS-B2 (2.5 μM) (B), U87MG cell control (C) and U87MG treated with DRS-B2 (2.5 μM) (D).

These differences of localization and aggregation of DRS-B2 on the plasma membrane of PC3 and U87MG cells raised the question of the cell viability after its binding. Therefore, the cell viability of the two cell lines was analyzed after 1h of treatment with DRS-B2 by flow cytometry. Cells were double labeled with FITC-Annexin-V (A-V) and 7-AAD. Fig 3 shows the amount of cells per staining in % of total cell number. As compare with untreated cells, when U87MG cells were treated with 2.5 μM DRS-B2, the percent of living cells (A-V^-^/7AAD^-^) was almost unaffected (from 90.3%+/- 1.25 to 84.3%+/- 2.97). When the same treatment was performed on PC3 cells, the percent of living cells shift from 85.1%+/- 2.1 in untreated cells to 58.1%+/- 6.36. In parallel, the amount of staining A-V^+^/7-AAD^-^ and A-V^+^/7-AAD^+^ indicates that PC3 cells undergo respectively in apoptosis and altered into the late apoptotic/necrotic state. The detail results are summarized in Table 3. These differences in labeling between the two cell lines were in accord with confocal microscopy results.

**Table 3:** Mean amount of cells per staining in % extracted from Fig 3. PC3 or U87MG cells were treated with DRS B2 (2.5 μM). One hour after treatment cells where double stained with FITC-Annexin-V and 7-AAD and analyzed by flow cytometry. The cell viability of cells was observed by measuring the amount of Annexin-V and 7-AAD negative and positive cells. Results represent the mean ± SEM of 3 determinations of A-V^-^/7-AAD^-^ = FITC-Annexin-V and 7-ADD negative; A-V^-^/7-AAD^+^ = FITC-Annexin-V negative and 7-AAD positive; A-V^+^/7-AAD^-^ = FITC-Annexin-V positive and 7-AAD negative; A-V^+^/7-AAD^+^ = FITC-Annexin-V and 7-AAD positive.

Tumor Cells	Percentage of cells staining (mean ± sem)			
	PC3			
Staining	A-V^-^/ 7AAD^-^	A-V^-^/ 7AAD^+^	A-V^+^/7AAD^-^	A-V^+^/7AAD^+^
Control	85.10 ± 2.10	0.51 **±** 0.20	7.04 **±** 0.30	7.32 **±** 0.51
DRS-B2 (2.5 μM)	58.10 **±** 6.36	0.46 **±** 0.30	21.95 **±** 3.46	19.55 **±** 2.90
**Tumor Cells**	**U 87MG**			
Staining	A-V^-^/ 7AAD^-^	A-V^-^/ 7AAD^+^	A-V^+^/7AAD^-^	A-V^+^/7AAD^+^
Control	90.30 **±** 1.25	0.20 **±** 0.15	5.61 **±** 0.20	3.91 **±** 0.15
DRS-B2 (2.5 μM)	84.30 **±** 2.97	0.18 **±** 0.08	7.98 **±** 1.18	7.55 **±** 2.85

### Identification of molecular partners of DRS-B2 to the membrane surface of susceptible cells

Given the results of the localization of DRS-B2 on the cell membrane of the PC3 cells, we considered the existence of molecular partners of the peptide. To verify this hypothesis, an N-Biotin-DRS-B2 was synthesized to study its direct interaction with the cellular cancer cell models. This DRS-B2 analog displayed the same antiproliferative activity on PC3 cells as DRS-B2.

#### Study of the interaction of DRS-B2 on PC3 by cell-based ELISA

**Fig 4:**
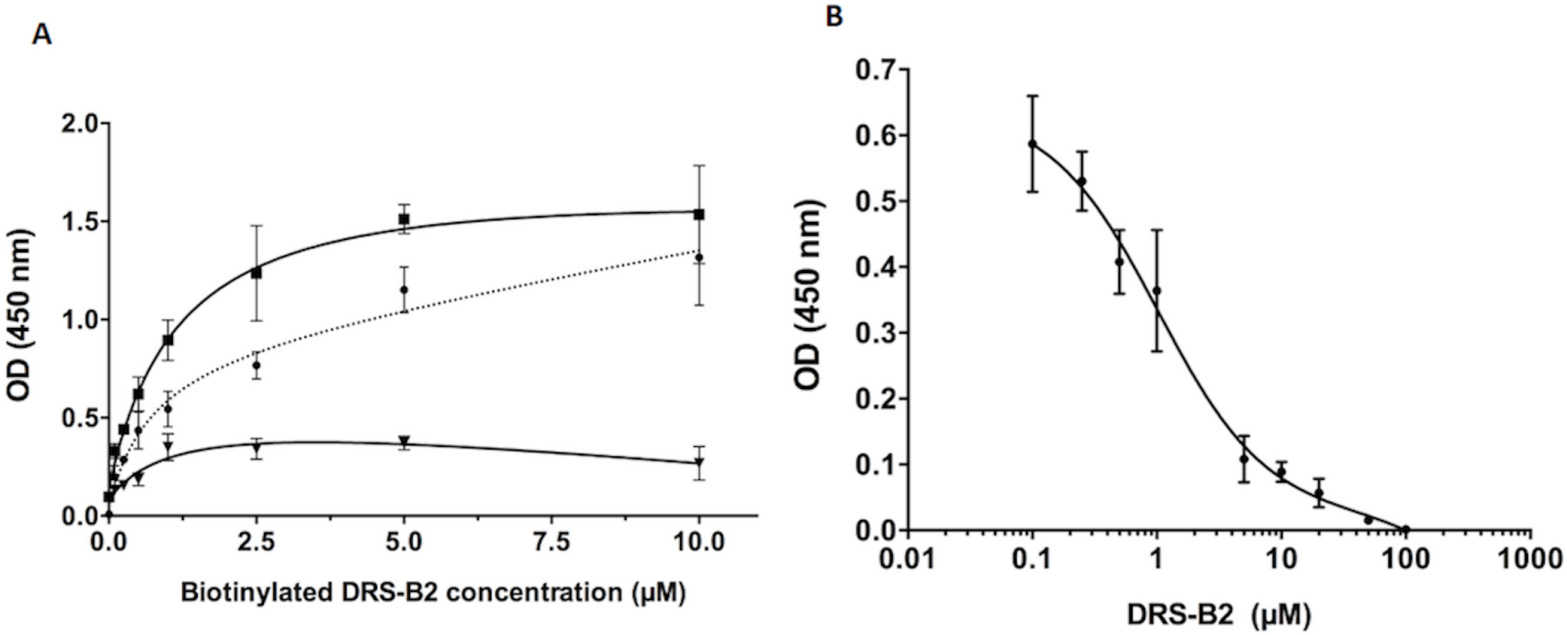
**(A) Saturation binding curves of increasing concentration of biotinylated DRS-B2 on PC3 cells**. In the presence (black triangles) or absence (black squares) of excess DRS-B2 (100 times more concentrated) with representation of the resulting specific binding (black dots). **(B) Inhibition of biotinylated DRS-B2 (1 μM) binding to PC3 cells with increasing concentrations of DRS-B2**.

Fig 4A demonstrates that the binding of biotinylated DRS-B2 on PC3 cells is dose-dependent and saturable with a calculated affinity constant Kd of 1.5 μM. This value is in agreement with the peptide concentration inhibiting 50% of cell growth as described previously [13]. To support this result, competition experiments were performed using non-biotinylated DRS-B2 as competitor on PC3 cells. As shown in Fig 4B, DRS-B2 inhibited binding of biot-DRS-B2 with a calculated inhibition constant (Ki) of 1.3 μM [18]. This concentration is consistent with the Kd value and could be explained by the fact that the peptide and its biotinylated analog bind to the cells with identical affinities.

#### Searching for protein partners

**"Pull down" experiments** The search for a protein partner was initiated by incubating 1.5 mg of total protein extract of human prostate PC3 tumor cells with streptavidin-Sepharose beads coupled to N-Biotin-DRS-B2. In the control experiment the same amount of protein extract was incubated with streptavidin-Sepharose beads alone. No protein partners were identified through the pull down experiments.

**Comparison of the antiproliferative effect of [all. D]-DRS-B2 vs DRS-B2**. We therefore investigated the implication of non-protein binding partners such as lipids and glycosaminoglycans. Furthermore, we hypothesized that the low activity of DRS-B2 in U87MG cells compared to its high activity in PC3 cells *in vitro* could be due to a difference in protease secretion by the two cell lines. As a consequence, we synthesized an analog of DRS-B2 designated [all. D]-DRS-B2 containing all the amino acids of the original peptide in their D-configuration. This change resulted in a modification of the secondary-tertiary structure (from α-helical "left" to α-helical "right") known to have greater resistance to proteases without affecting its biological activity [19].

**Fig 5:**
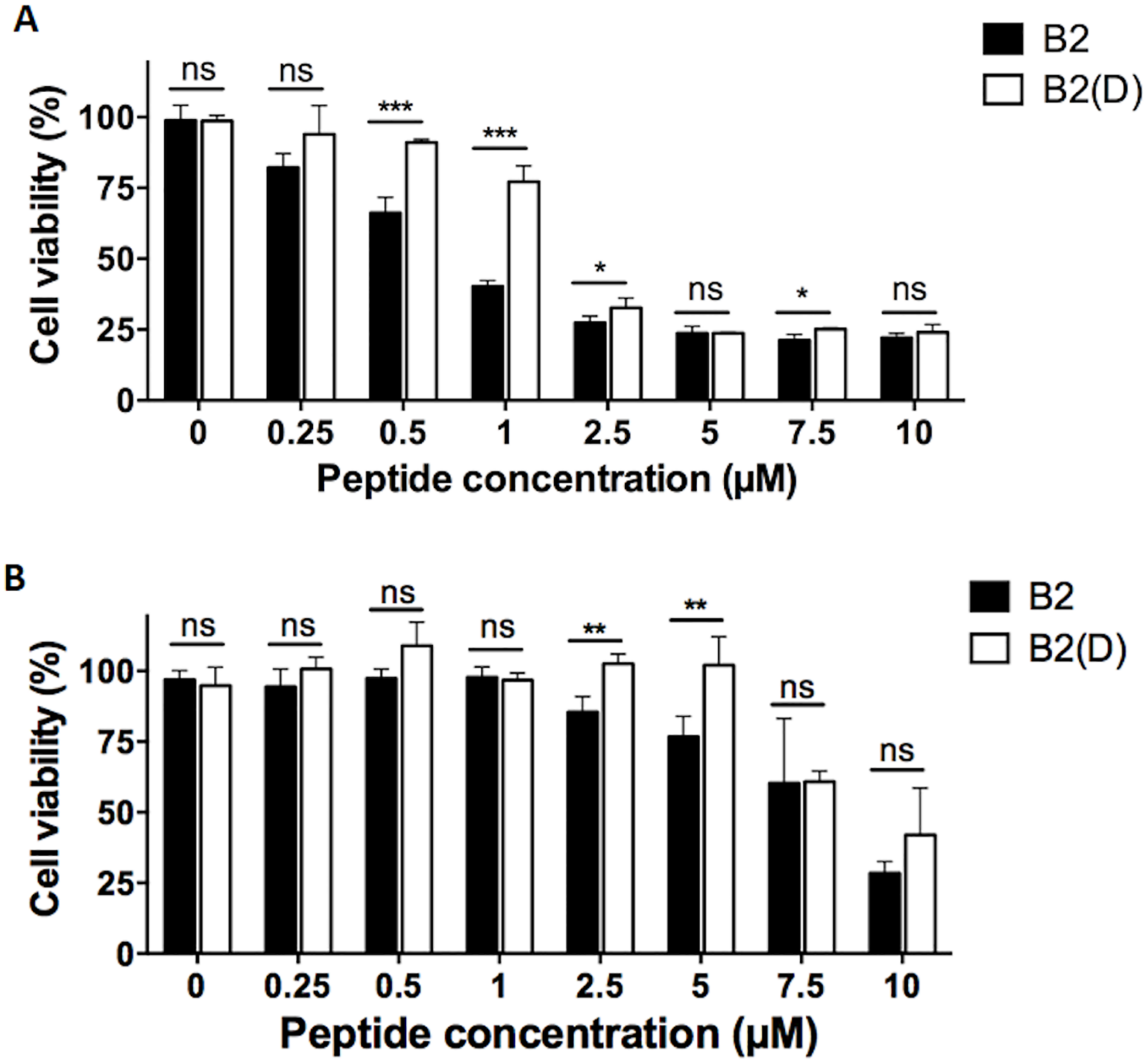
**(A) *In vitro* anti-proliferative effect of PC3 cells**. In presence of DRS-B2 (black bars) or in presence of its analog [all. D]-DRS-B2 (white bars). On the first and third day after plating, the cells were treated with different concentrations of peptides. Twenty-four hours after the last treatment cell counting was performed with crystal violet staining. Results are expressed in percent of cell viability per well. Results represent the mean ± SEM of three determinations. * p < 0.05; ** p < 0.01; *** p < 0.001 versus control (untreated cells). **(B) *In vitro* anti-proliferative effect of U87MG cells**. In presence of DRS-B2 (black bars) or in presence of its analog [all. D]-DRS-B2 (white bars). Cells were treated as described for Fig 5A.

The results showed that the change in configuration had a low impact on the peptide activity in the *in vitro* proliferation of PC3 or U87MG cells and did not lead to the conclusion that there was a stereospecific interaction but they confirms that DRS-B2 is as stable as its analog [all. D]-DRS-B2 in both cell lines (Fig 5). This result does not support the hypothesis of the existence of a specific protein receptor for DRS-B2 since it is commonly accepted that the interaction of a ligand with its protein receptor is usually stereo-selective [19].

#### Searching for glycomic partners

Numerous studies have shown that the membranes of cancer cells typically carry a net negative charge due to overexpression of anionic molecules at their surface. These molecules include: phosphatidylserine, sialic acid (N-acetylneuraminic acid) and glycosaminoglycans (GAGs) [3,20–22]. The two major classes of GAGs present at the surface of membranes are chondroitin sulfate (CS) and heparan sulfate (HS). Both are sulfated and could represent the potential anionic targets of cationic peptides such as the LfcinB and other lytic peptides [23, 24]. The nature of CAPs, as is for DRS-B2, implies susceptibility to interact with GAGs through negative charges carried by sulfates. We then investigated if GAGs could interfere in the cytotoxic activity of DRS-B2.

**Effect of DRS-B2 on the proliferation of PC3 cells treated or not with sodium chlorate (NaClO**_**3**_). To investigate the implication of the negative charges carried by the sulfated GAGs on the cytotoxic activity of DRS-B2 we used sodium chlorate (NaClO_3_) whose effect is to reduce sulfatation by inhibiting ATP sulfurylase [25].

**Fig 6:**
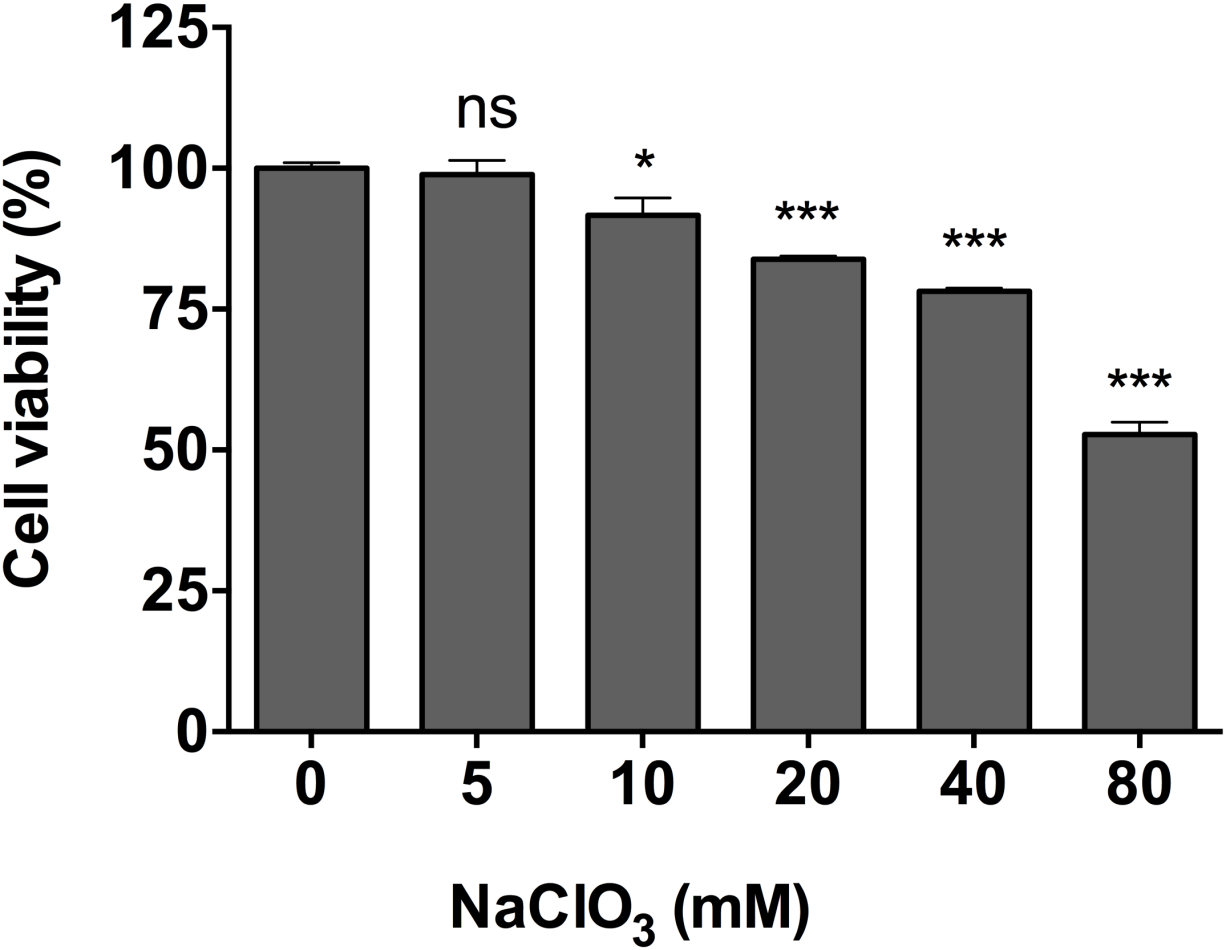
Percentage of living PC3 cells in the presence of different concentrations of sodium chlorate (0–80 mM). PC3 cells were first seeded in a 24-well plate as previously described and on the second day of incubation, increasing concentrations of sodium chlorate (0 to 80 mM) were added and crystal violet staining was performed on the fourth day. Results are expressed in percent of cell viability per well. Results represent the mean ± SEM of three determinations. * p < 0.05; ** p < 0.01; *** p < 0.001 versus control (untreated cells).

**Fig 7:**
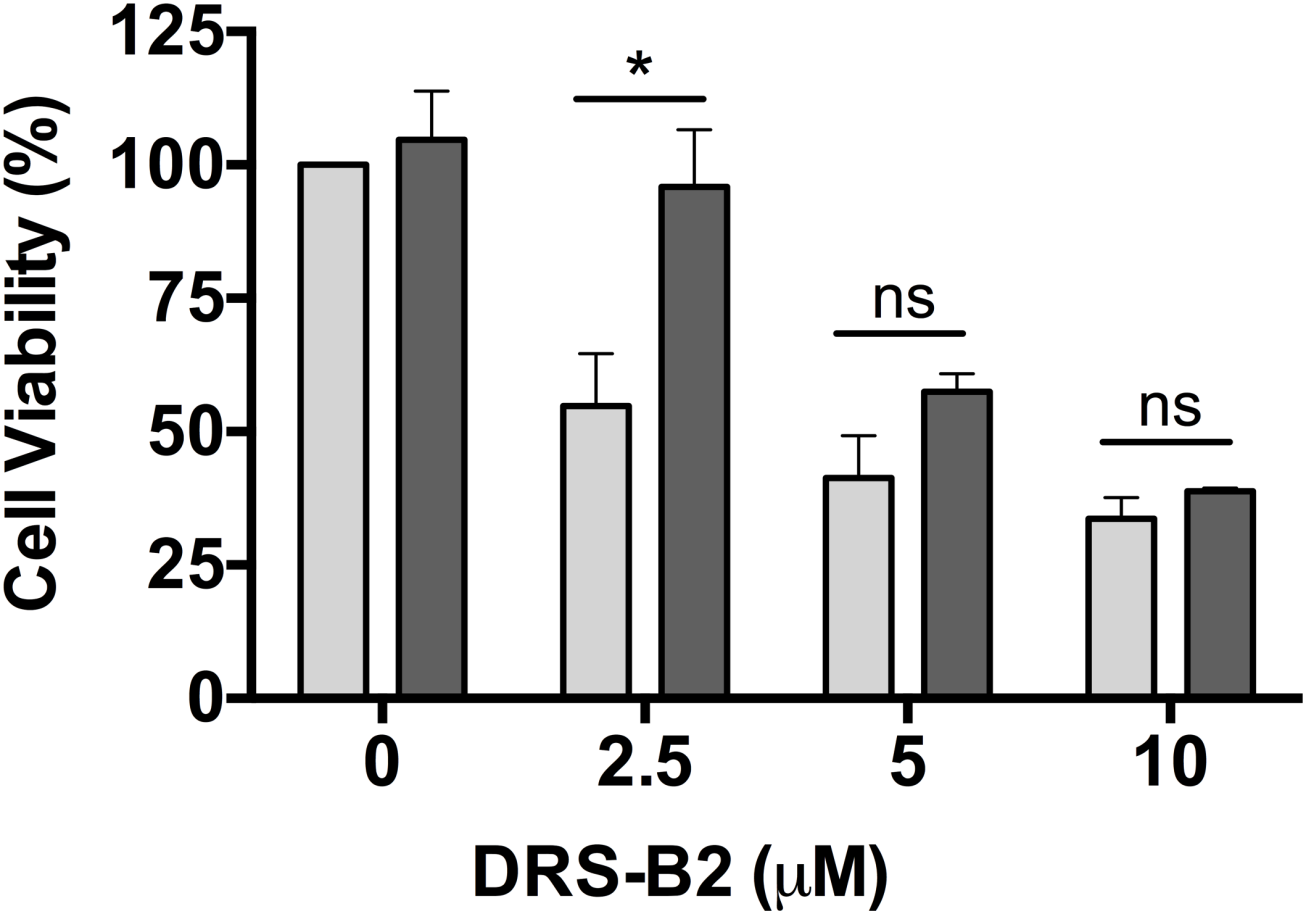
Percentage of living PC3 cells in presence of DRS-B2 and sodium chlorate. Cells were seeded and treated on the second day with 10 mM sodium chlorate as described for Fig 6. Experiments were performed in the absence or presence of 3 different concentrations of DRS-B2 (2.5, 5 or 10 μM) in the absence (light gray bars) or presence (dark gray bars) of sodium chlorate (10 mM) crystal violet staining was performed on the fourth day after seeding. Results are expressed in percent of cell viability per well. Results represent the mean ± SEM of three determinations. * p < 0.05; ** p < 0.01; *** p < 0.001 versus control (untreated cells).

First, incubation of PC3 cells in the presence of increasing concentrations of NaClO_3_ (0–80 mM) allowed us to determine the minimal toxic concentration that could be used, which was fixed at 10 mM and used in the remainder of our study (Fig 6). Secondly, the incubation of PC3 cells with increasing concentrations of DRS-B2 showed that the antiproliferative effect of DRS-B2 at 2.5 μM was abolished when cells were pre-treated with 10 mM NaClO_3_ (Fig 7). This suggested that sulfated-GAGs could be partially involved in the mechanism of action of DRS-B2.

**Fig 8:**
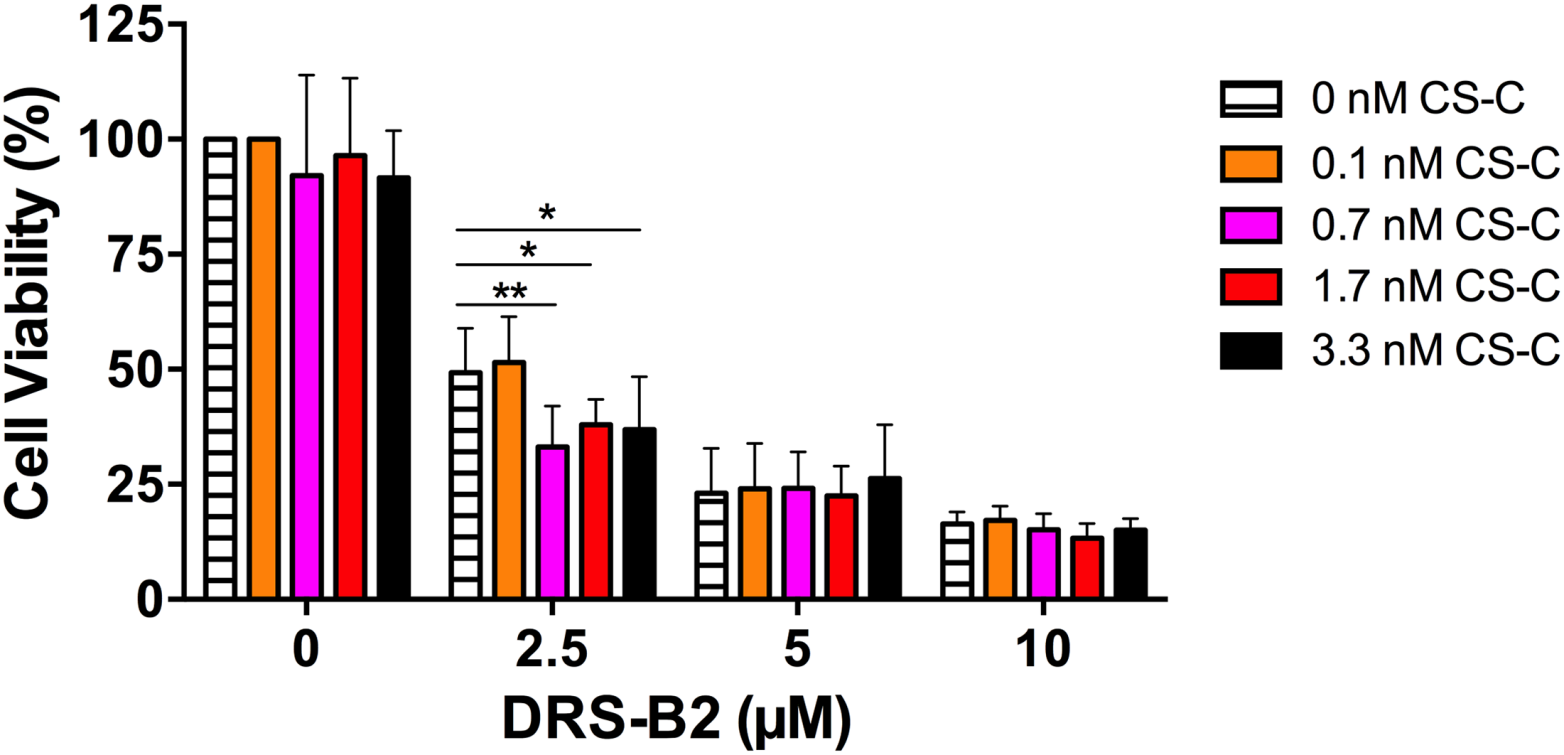
Percentage of living PC3 cells in presence of DRS-B2 and CS-C. Cells were seeded as previously described in Fig 5A; experiments were performed in presence of different concentrations of CS-C (0–3.3 nM) alone or in presence of DRS-B2 at different concentrations (0–10 μM). Crystal violet staining was performed on the fourth day after seeding. Results are expressed in percent of cell viability per well. Results represent the mean ± SEM of three determinations. * p < 0.05; ** p < 0.01; *** p < 0.001 versus control (untreated cells).

**Study of the antiproliferative activity of DRS-B2 on PC3 cells in the presence and absence of chondroitin sulfate C (CS-C)**. PC3 cells were incubated with three different concentrations of DRS-B2 (2.5, 5 or 10 μM) corresponding to its GI_50_, 2 GI_50_ and 4 GI_50_, respectively and increasing concentrations of CS-C (0 to 3.3 nM) (Fig 8). The effect of CS-C was subtracted to represent the effect of DRS-B2. The results showed that in the absence of CS-C the percentage of living cells decreased from 49% to 23% and 16% when the cells were incubated in the presence of DRS-B2 at 2.5, 5 and 10 μM respectively (Fig 8). In these conditions the cytotoxic effect of DRS-B2 is dose dependent. When this experiment was repeated in the presence of the 3 different concentrations of DRS-B2 and an increasing concentration of CS-C, the percentage of living cells was unaffected in the presence of 5 or 10 μM DRS-B2. Under these conditions, CS-C seemed to have no effect on the cytotoxic activity of the peptide, whereas at 2.5 μM DRS-B2, the percentage of living cells dropped from 49% to 33% in the presence of 0.7 nM CS-C. These results allow us to conclude that CS-C at very low concentrations (0.7 nM) potentiates the inhibitory effect of DRS-B2 when used at concentration near its GI_50_.

**Fig 9:**
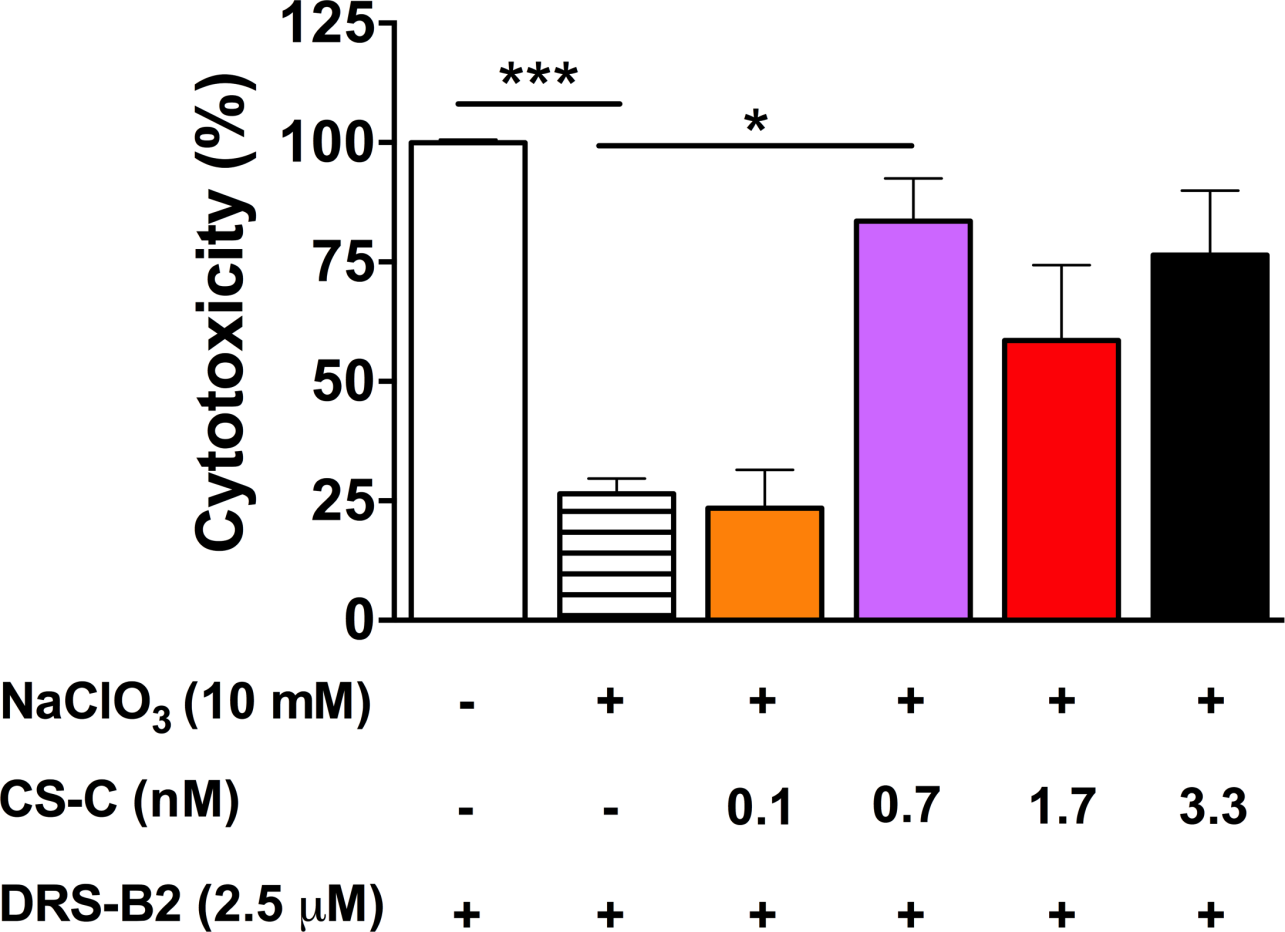
Percentage of cytotoxicity of PC3 cells in presence of DRS-B2, sodium chlorate and CS-C. Experiments were performed in presence of DRS-B2 (2.5 μM) alone or in the presence of sodium chlorate (10 mM) and with or without different concentrations of CS-C (0–3.3 nM). LDH release was measured with CytoTox 96 kit 3 hours after treatment. Results are expressed in percentage of cytotoxicity versus time of treatment (see Materials and methods section). Results represent the mean ± SEM of 3 determinations. * p < 0.05; ** p < 0.01 ***; p < 0.001 versus treatment with DRS-B2 alone.

**Measurement of cytoplasmic LDH-release by PC3 cells in the presence of DRS-B2 with or without sodium chlorate and various CS-C concentrations**. In this experiment, the results were compared with the percentage of cytotoxicity of 2.5 μM DRS-B2 on PC3 wells, defined as 100% cytotoxicity (Fig 9). In the presence of 10 mM sodium chlorate the cytotoxic effect of 2.5 μM DRS-B2 dropped to 25% (Fig 9). This confirmed that sodium chlorate inhibited the activity of DRS-B2 on PC3 cells.

Under the same conditions, addition of increasing concentrations of CS-C (0–3.3 nM) partially reversed the sodium chlorate inhibitory effect without reaching 100% induced by DRS-B2 alone.

All these results support the hypothesis of the involvement of CS-C in the anti-proliferative mechanism of action of DRS-B2 but its exact role still remains unclear. We know that the α helix structure of DRS-B2 in an environment mimicking the membrane is a key element in its antibacterial activity. As DRS-B2 appears to interact with GAGs through negative charges carried by sulfates, it is legitimate to question the influence of GAGS on the structure of DRS-B2. To test this possibility, we studied on one hand the influence of environmental changes on the tryptophan (W) residue by measuring the fluorescence emission of this amino acid in the presence and absence of CS-C, and on the other hand, the structural changes of DRS-B2 by circular dichroism (CD) in the same experimental conditions.

### Structure activity relationships

#### Influence of CS-C on the structure of the DRS-B2

**Fig 10:**
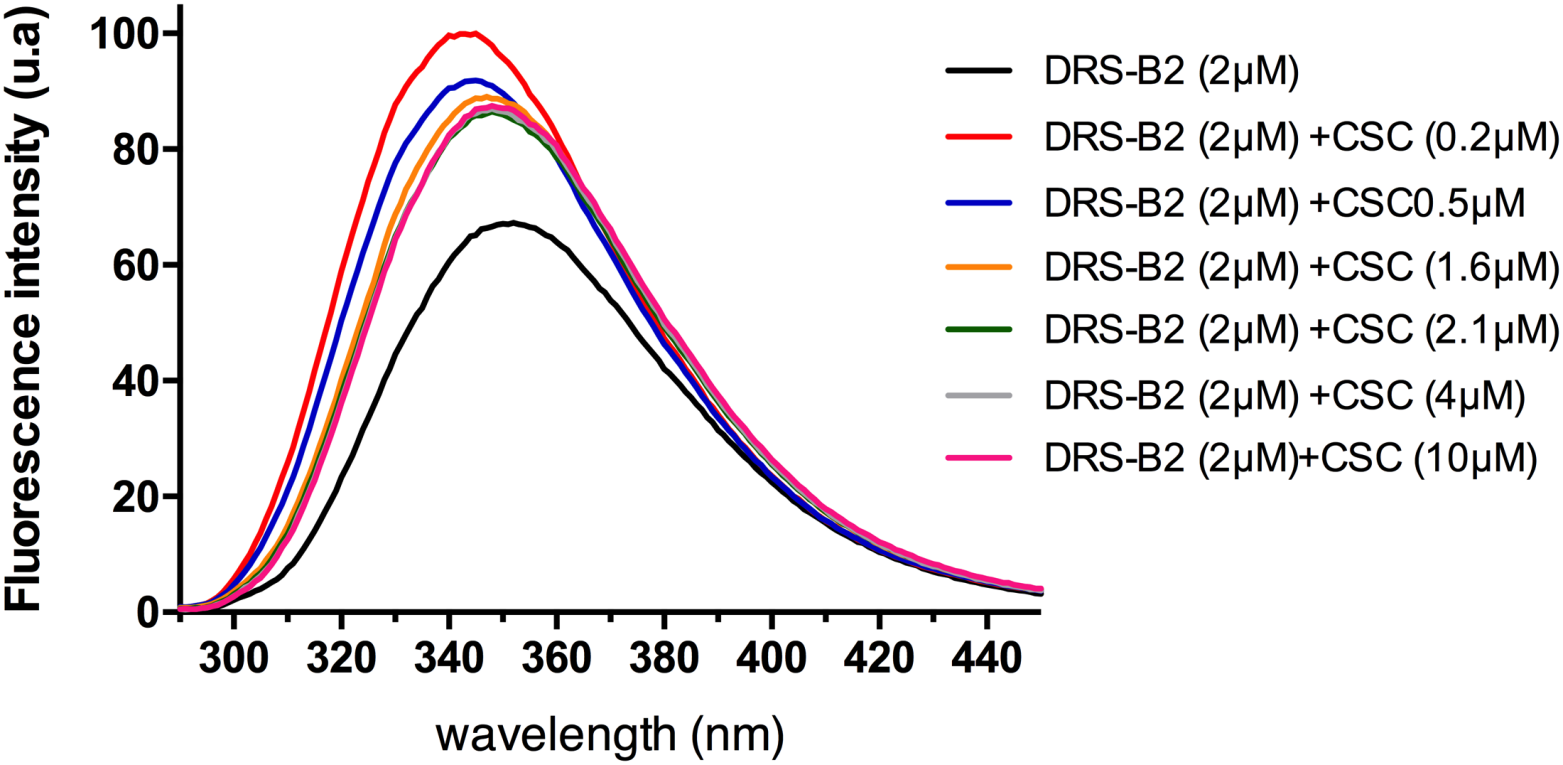
Fluorescence emission spectra of Trp of DRS-B2 (2 μM). In the absence or in the presence of increasing concentrations of CS-C (0.2–10 μM).

When the W residue present in position 3 of the DRS-B2 sequence was excited at 280 nm, an emission of the fluorescence with a characteristic peak at 340 nm (Fig 10) was detected. The fluorescence intensity of DRS-B2 in the presence of a low concentration of CS-C (0.2 μM) showed a shift to shorter wavelengths (hypsochromic effect) and an increase in the intensity of the fluorescence (hyperchromic effect) (Fig 10). By adding concentrations of CS-C from 0.5 μM to 10 μM we observed a shift of the wavelength measured with DRS-B2 alone to the left, and a decrease in the intensity of fluorescence that nevertheless remained higher than that detected with DRS-B2 alone (Fig 10). The increase in peak intensity reflects an increase in the molar epsilon that is directly related to the absorbance. Overall, the hypsochromic effect observed at low concentrations of CS-C allows us to hypothesize the burial of the W residue within the structure of the peptide that becomes unavailable to the solvent, and to re-exposure of the W residue when the concentration of CS-C increases. Two possibilities may explain these observations:

(i) Either the W is hidden by polymers of CS-C,
(ii) or a structural change in the DRS-B2 in the presence of CS-C leads to the burial of W.

To test these possibilities and explain why only low concentrations of CS-C favor structuring of DRS-B2, we examined the structure of DRS-B2 in the absence and in the presence of the CS-C by CD.

#### Study of the structure of the DRS-B2 by CD

**Fig 11:**
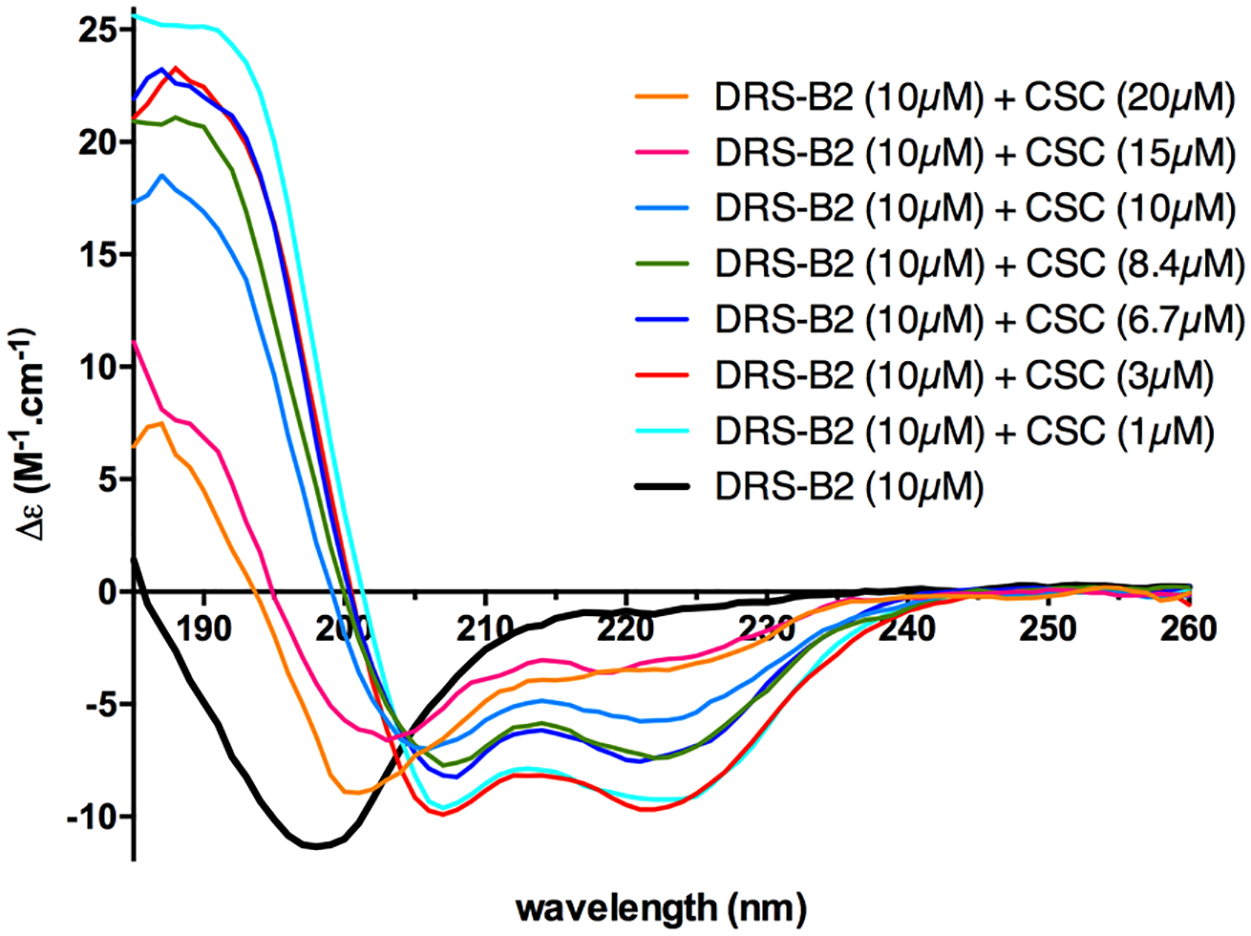
Circular dichroism spectrum of the DRS-B2 (10 μM). In water or in the presence of increasing concentration of CS-C: (1–20 μM).

In Fig 11 are shown the CD spectra of DRS-B2 in water or in the presence of increasing CS-C concentrations. While DRS-B2 in water presented no structure, the addition of small amounts of CS-C (1–3 μM) in the absorption spectrum, led to two minima at wavelengths 208 and 222 nm, that are the characteristics of an α-helical structure. The maximum percentage of helix of DRS-B2 in the presence of CS-C occurs at 1 μM CS-C and then decreases when the CS-C concentration further increases. An almost total destructuration of the helix is visible beyond 10 μM -15 μM of CS-C.

This finding raises the question of the relationship that may exist between the potentiating effect of the DRS-B2 cytotoxic activity and its α-helix structure observed at low CS-C concentrations.

#### Study of antiproliferative activity of [W/F]^3^DRS-B2 and DRS-B2 on PC3 cells

To determine the role and involvement of the W residue in the anti-proliferative activity of the DRS-B2 in relation with a potential interaction with GAGs, we synthesized DRS-B2 and its analog in which the W residue in position 3 was substituted by a phenylalanine (F) residue ([W/F]^3^DRS-B2). We studied the antiproliferative activities of these peptides and their secondary structure by CD in presence or absence of CS-C.

**Fig 12:**
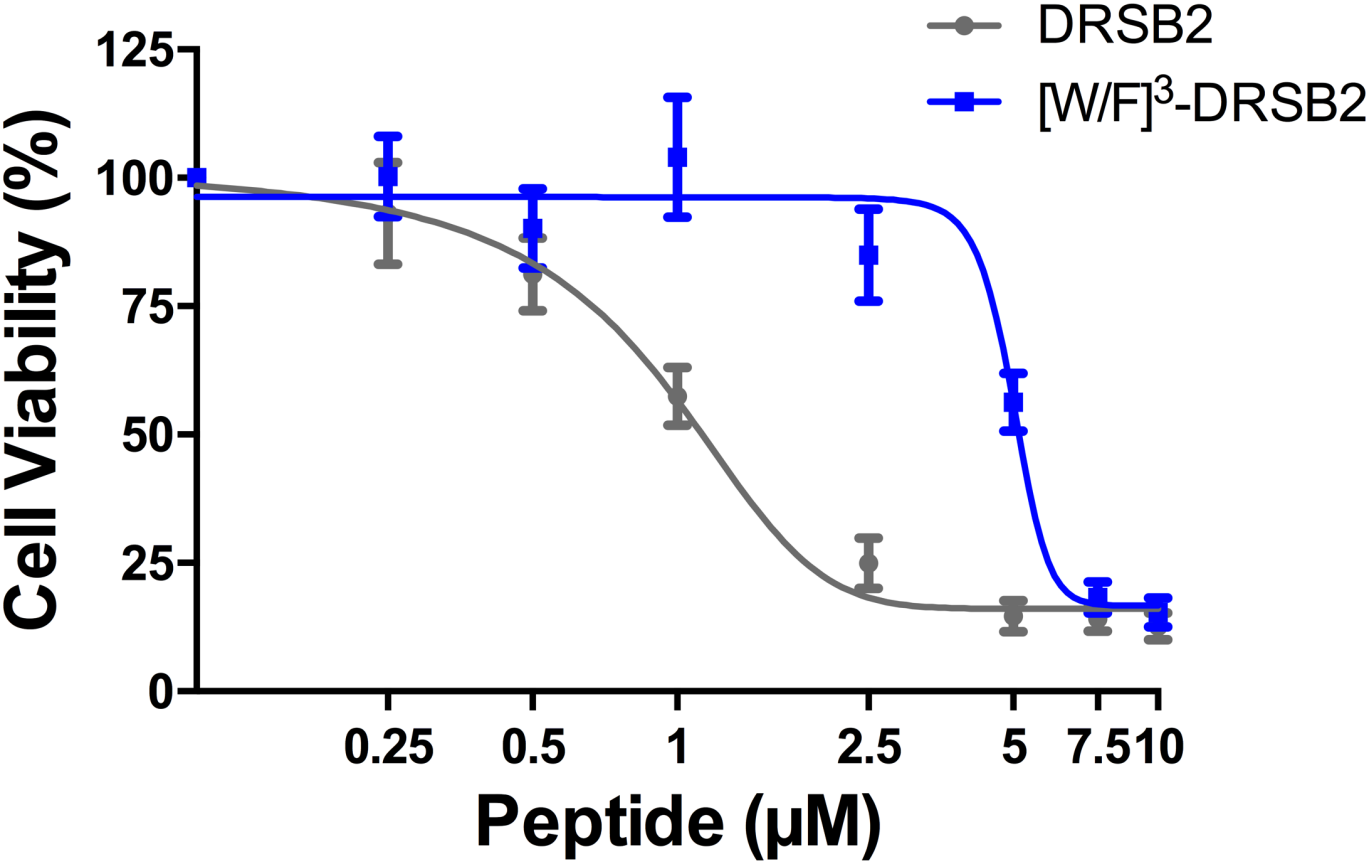
Effect of increasing concentrations of DRS-B2 or [W/F]^3^-DRS-B2 on proliferation of PC3 cells. On the first and third day after plating, the cells were treated with different concentrations of peptides. Twenty-four hours after the last treatment cell counting was performed with crystal violet staining. Results are expressed in percent of cell viability per well. Results represent the mean ± SEM of three determinations. * p < 0.05; ** p < 0.01; *** p < 0.001 versus control (untreated cells).

Previously we showed that PC3 cells are sensitive to DRS-B2. Therefore, these cells were used to study the impact of the substitution of the W residue in position 3 on the anti-proliferative activity of these two peptides. Fig 12 shows the results obtained, expressed as percentage of living cells versus the increasing concentration of peptides. We observed a dose-dependent anti-proliferative activity of DRS-B2 that was 5 times more efficient than [W/F]^3^DRS-B2. Indeed, the concentration of modified peptide required to cause 50% inhibition of cell proliferation was 5 μM to achieve the same effect as the DRS-B2 (Table 4).

**Table 4:** Concentration of DRS-B2 and [W/F]^3^-DRS-B2 that inhibits 50% of cell growth (GI_50_) extracted from Fig 12.

Peptides	GI_50_ (μM ± sem)
DRS-B2	0.86 ± 0.14
[W/F]^3^-DRS-B2	4.99 ± 0.18

We can conclude that the W residue is essential for the optimal anti-proliferative activity of DRS-B2. In addition, the involvement of the W residue in the interaction of cationic peptides such as cell penetrating peptides (CPPs) with GAGs has been described and suggests that the W residue of DRS-B2 could play a key role in the antiproliferative activity of this peptide. As described in the present study, sulfated GAGs, such as CS-C, seem essential for DRS-B2 antiproliferative activity. The activity of DRS-B2 and [W/F]^3^DRS-B2 on PC3 cells in the presence or absence of the CS-C was therefore examined.

#### Antiproliferative activity of the peptides in the presence of CS-C

PC3 cells were treated with increasing concentrations of CS-C that had been pre-incubated with each of the two peptides at concentrations near their previously determined GI_50_, 1 μM and 5 μM for DRS-B2 and [W/F]^3^DRS-B2, respectively.

**Fig 13:**
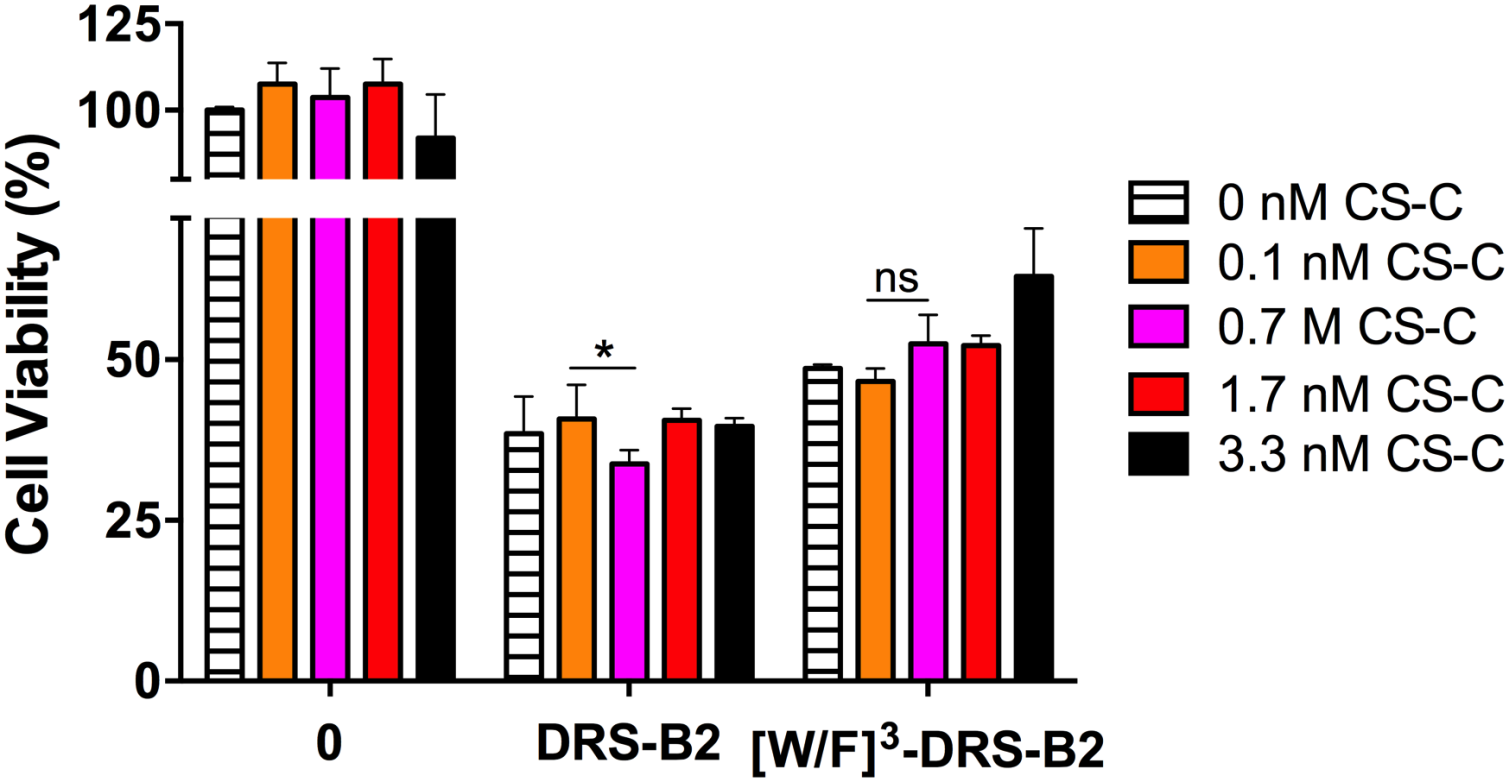
Percentage of living PC3 cells in the presence of different concentrations of CS-C (0–3.3 nM). Experiments were performed without peptide or in the presence of DRS-B2 (1 μM) or [W/F]^3^DRS-B2 (5 μM). On the first and third day after plating, the cells were treated with or without peptides and different concentrations of CS-C. Twenty-four hours after the last treatment cell counting was performed with crystal violet staining. Results are expressed in percent of cell viability per well. Results represent the mean ± SEM of three determinations. * p < 0.05; ** p < 0.01; *** p < 0.001 versus control (untreated cells).

The results reported in Fig 13, representing the percentage of living cells based on the concentration of CS-C in the absence or presence of the peptides showed that CS-C has a low potentiating effect on the anti-proliferative activity of DRS-B2. Indeed, the number of PC3 cells pre-treated with DRS-B2 (1 μM) in presence of CS-C (0.7 nM), decreased by about 15%, whilst there was an increase of about 10% in the number of such pre-treated cells by [W/F]^3^DRS-B2 (5 μM) in the presence of the same concentration of CS-C, indicating a slight inhibition of the anti-proliferative activity of this analog.

We can conclude first, that CS-C (0.7 nM) potentiates the anti-proliferative effect of DRS-B2 and inhibits that of [W/F]^3^DRS-B2, and secondly, that it highlights the importance of the W residue in the anti-proliferative activity of DRS-B2.

#### Study of the structure [W/F]^3^DRS-B2 by CD

The secondary structure of DRS-B2 has already been completed by CD analyses in the absence and presence of CS-C that induces a maximum α-helical structure when this GAG is added at 1 μM.

We also investigated the secondary structure of its analogue, [W/F]^3^DRS-B2 at a concentration of 10 μM in the presence of increasing concentrations of CS. The results indicated that [W/F]^3^DRS-B2 in water did not exhibit the structure, but adopted an α-helical structure when the concentration of 1 μM CS-C was reached. The percentage of this α-helical structure decreased when the concentration of CS-C increased. Beyond 10 μM, we witnessed an almost total breakdown of the structure of the DRS-B2- analog. However, under the same analysis conditions, the percentage of α-helical structure for DRS-B2 (89.5%) in the presence of 1 μM of CS-C is 4 times greater than the one for [W/F]^3^DRS-B2 (20.3%) (Table 5)

**Table 5:** Quantitative analysis of DRS-B2 and [W/F]^3^-DRS-B2 in a Far UV circular dichroism spectra by Neural Networks (16).

	DRS-B2(10 μM)	DRS-B2 (10 μM) + CS-C (1 μM)	[W/F]^3^-DRS-B2 (10 μM)	[W/F]^3^-DRS-B2 (10 μM) + CS-C (1 μM)
**Helix**	07.5%	89.5%	12%	20.3%
**Antiparallel**	54%	00%	37.2%	24%
**Parallel**	06.5%	0.01%	08.6%	09%
**Beta-Turn**	17.8%	0.07%	16.2%	17.2%
**Random. Coil**	14.1%	0.03%	26%	30%

## Discussion

In this study we have focused on exploring the antitumor mechanism of action of DRS-B2 and especially on deciphering how it interacts with the plasma membrane so as to identify molecular partners present at the surface of the targeted tumor cells. First, we evaluated the anti-proliferative effect of DRS-B2 at different concentrations on fifteen human tumor cell lines including mammary carcinoma lines, prostatic adenocarcinomas, glioblastomas, pancreatic carcinomas and melanomas as well as non-tumor cells of human origin. These experiments showed that DRS-B2 inhibits cell proliferation in a dose-dependent manner in certain tumor lines. DRS-B2 acts mainly on cell prostate carcinomas (PC3, DU145) with low GI_50_ values (in the micromolar range), and shows poor effect on a glioblastoma cell line (U87MG) and no effect on healthy primary cells of human origin. These two cell lines PC3 and U87MG were chosen for further studies.

The use of polyclonal antibodies against DRS-B2 and confocal microscopy analyses showed a rapid aggregation of the peptide at the plasma membrane of PC3 tumor cells, then its penetration into the cells and into the nucleus [14]. However, if the early stages of the peptide-membrane interactions are well studied and seem widely accepted, the question related to the mechanism of internalization of the peptide remains to be answered. The synthesis and availability of the fluorescent Alexa594-(Cys^0^)-DRS-B2 allowed us to conduct the immune-localization studies on two tumor lines, PC3 that is sensitive to DRS-B2 and U87MG that presents resistance to DRS-B2.

This study has allowed us to show that DRS-B2 quickly accumulates at the cytoplasmic membrane of susceptible cells (PC3), and enters the cytoplasm and the nucleus. These observations are consistent with our previous results [14], but it would be interesting to carry out the same experiments at 4°C to determine if the peptide penetration could be a consequence of an endocytic mechanism. Unlike in PC3 cells, DRS-B2 is found packaged on the outside of the U87MG membrane and is not observed at the transmembrane level. These results suggest that the resistance of U87MG cells to DRS-B2 may be the result of the internalization mechanism, which appears to be different from that in PC3 cells. Indeed, the sequestration of DRS-B2 on the outer side of the cells of U87MG could be responsible for the inhibition of its membranotropic activity and/or its degradation by proteases that are co-localized in the sequestration vesicles. Flow cytometry results corroborated these observations and indicated that DRS-B2 treatment for one hour induced apoptotic and/or necrotic-like pathway more important in PC3 cells than in U87MG cells.

The aggregation and the specific localization of the DRS-B2 to the membrane of tumor cells mentioned above suggest the existence of molecular partners present at the surface of target cells. To test this hypothesis, we synthesized the biotin-N-DRS-B2 and studied its interaction with tumor cell lines that are sensitive to the DRS-B2. We first verified that this analog had the same anti-proliferative activity towards PC3 cells as DRS-B2. Although we showed the existence of a specific, saturable and reversible interaction of this analog with the membrane of the tumor cells studied, the "pull down" experiments did not confirm the existence of a protein partner localized at the surface of the plasma membrane or in the cytoplasm of these tumor cells. In addition, we showed that the anti-proliferative activity of DRS-B2 composed only of amino acids in the D configuration and tested on PC3 and U87MG cells is nearly identical to that of DRS-B2. These results confirm first, the absence of a stereo-selective receptor protein and second, the stability of DRS-B2 with respect to proteases.

These results prompted us to look for other kinds of partners than proteins. Several studies have shown that CAPs also kill certain cancer cells but do not appear to destroy healthy mammalian cells [26]. In this respect, the electrostatic interactions between the cationic peptides and anionic components of cell membranes are considered as major factors in the selective destruction of cancer cells. The membranes of cancer cells usually have a net negative charge due to a higher than normal expression of anionic molecules such as phosphatidylserine (<9% of total phospholipids of membranes) and of O-glycosylated mucins, or alterations in the carbohydrate portion of glycoproteins and glycolipids including increased sialylation and the presence of GAGs, contributing to the electronegativity of the membranes of tumor cells [3, 22–23]. This overall charge difference on the membrane surface of healthy and tumor cells may partially explain the ability of CAPs to kill certain cancer cells while sparing healthy ones. To identify the binding target of DRS-B2, we first examined the effect of GAGs on the anti-proliferative activity of this peptide. Thus, we have shown that pretreatment of PC3 tumor cells with sodium chlorate, that is an inhibitor of sulphatation of GAGs, results in partial inhibition of the anti-proliferative activity induced by DRS-B2 and that this inhibition is reversed when the same experiment is performed in the presence of low CS-C concentrations. Furthermore, treatment of PC3 cells with DRS-B2 pre-incubated with increasing concentrations of CS-C potentiated the anti-proliferative activity of DRS-B2 by CS-C at low concentration. These results need to be confirmed and extended by studying other GAGS with anionic properties that could be potential targets of DRS-B2. Moreover, it would be interesting to determine the GAG distribution and composition at the tumor cell surface to demonstrate if a certain pattern of GAGs could be specifically implicated in DRS-B2 internalization. Indeed, several studies showed the participation of heparan sulfate proteoglycans in the interaction between arginine-rich CPPs and the cell membrane leading to cellular internalization [27–29].

Treatment with different concentrations of two analogs of DRS-B2, (1–23)-DRS-B2 and (24–33)-DRS-B2 corresponding respectively to the domain of the DRS-B2 structured in an α-helix and its hydrophobic domain, did not show significant inhibition on PC3 proliferation [14]. In addition, the combined treatment with (1–23)-DRS-B2 and (24–33)-DRS-B2 has no effect on PC3 proliferation. This demonstrates that a physical link between the two domains of DRS-B2 is important for the integrity of its anti-proliferative activity.

We also synthesized [W/F]^3^-DRS-B2 in which the residue W in the third position is replaced by an F residue. We have shown that the absence of W in the sequence of DRS-B2 decreases the anti-proliferative activity five fold. In view of these results, we examined if the cause of the analog’s decreased activity could be due to the absence of its interaction with GAGs. Indeed, recent studies have shown that increasing incorporation of W residues in the primary structure of cationic CPPs results in an increase of their affinity for GAGs [30]. In the present study, although the potentiating effect of CS-C is not pronounced with DRS-B2, it is interesting to note that the absence of W in the DRS-B2 analog seems to produce an opposite effect since the anti-proliferative effect of the modified peptide is inhibited by CS-C.

Knowing that the secondary structure of the CAPs is essential for biological activity, the last part of our study consisted in studying the influence of GAGs on the secondary structure of DRS-B2 and [W/F]^3^-DRS-B2 by measuring the fluorescence emission of the W residue and the CD spectra. The fluorescence emission of W at 350 nm in the absence and in the presence of increasing concentrations of CS-C provides some information on the environment of this pharmacophore as well as on the structural changes that DRS-B2 would undergo. In the presence of low concentrations of CS-C, there was an increase in intensity of the fluorescence signal (hyperchromic effect) and at the same time a shift towards shorter wavelengths (hypsochromic effect) with respect to the fluorescence emitted by DRS-B2 alone or in the presence of high concentrations of CS-C. These results suggest either a structural modification of DRS-B2 induced by CS-C resulting in burying of the W residue or direct interaction of CS-C with DRS-B2 that will mask the pharmacophore.

To further examine these two hypotheses, CD spectrum measurements of DRS-B2 in water and in the presence of increasing concentrations of CS-C were performed. The results show that DRS-B2 has a random structure or "random coil" in water, whereas it adopts an α-helical structure in the presence of low concentrations of CS-C (1 μM—10 μM). This structure disappears when the concentration of CS-C is greater than 10 μM. Therefore, CS-C probably would not be the direct target of DRS-B2 in its anti-proliferative action, but it would participate in the formation of the α-helix that is the bioactive structure. Moreover, the comparison of the secondary structure of DRS-B2 and of its analog [W/F]^3^DRS-B2 allowed us to show that the absence of W seems to impact on the formation of the α-helix structure in the presence of GAGs and would therefore be the cause of the decline in activity.

In conclusion, our results demonstrated that DRS-B2 interacts directly with the lipids of the plasma membrane and that the CS potentiates this activity by favoring probably its structuration in α-helix amphipathic structure and highlight the importance of the W residue in the anti-proliferative activity of DRS-B2. In addition, these cationic antimicrobial peptides appear to be more active on prostatic tumor cells and without effect on normal cells and certain other tumor cells. As a consequence, DRS-B2 represents an innovative technological platform for the development and design of new molecules usable to selective targeting of some tumor cells. Further investigations are needed to understand this selective effect on prostatic tumor cells.

## Supporting information

S1 Fig Purification of (Cys^0^)-DRS-B2 by HPLC. (left ordinate: Absorbance at 220 nm in arbitrary unit; right ordinate: percentage of acetonitrile).
(TIFF)

S2 Fig Maldi-Tof mass analysis. (TIFF)

S3 Fig Purification of [Alexa594]-(Cys^0^)-DRS-B2 by size chromatography exclusion. (TIFF)

S4 Fig Effect of increasing concentrations of D+S-B2 on the proliferation of diverse tumor cells. (TIFF)
