# Supplementary figures and images for: Studies of the antitumor mechanism of action of dermaseptin B2, a multifunctional cationic antimicrobial peptide, reveal a partial implication of cell surface glycosaminoglycans
Source: PLoS One. 2017 Aug 10;12(8):e0182926. doi: 10.1371/journal.pone.0182926 (PMC5552233; doi:10.1371/journal.pone.0182926)

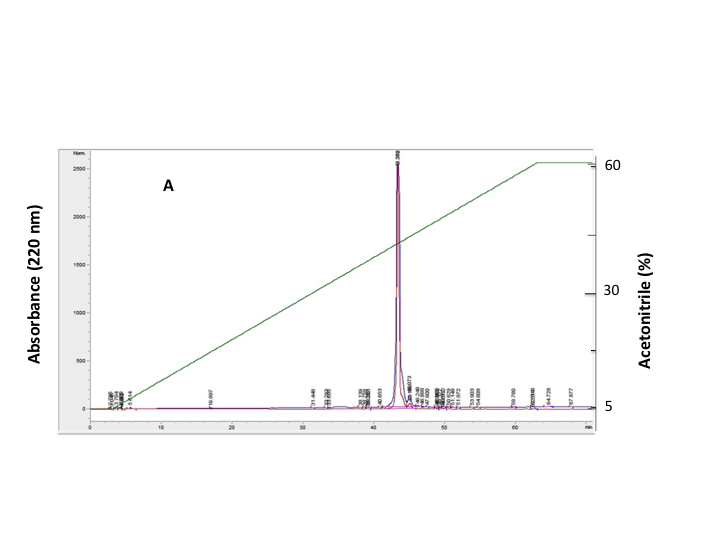

Supplement: S1 Fig — (left ordinate: Absorbance at 220 nm in arbitrary unit; right ordinate: percentage of acetonitrile). (TIFF) [file pone.0182926.s001.tiff]

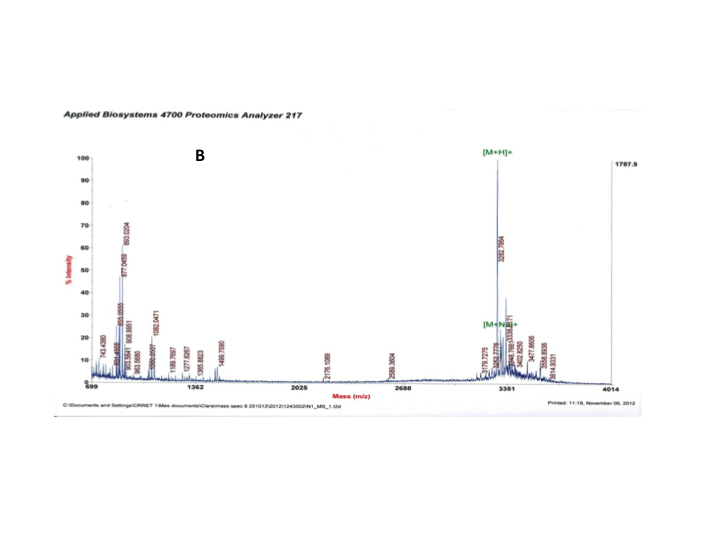

Supplement: S2 Fig — (TIFF) [file pone.0182926.s002.tiff]

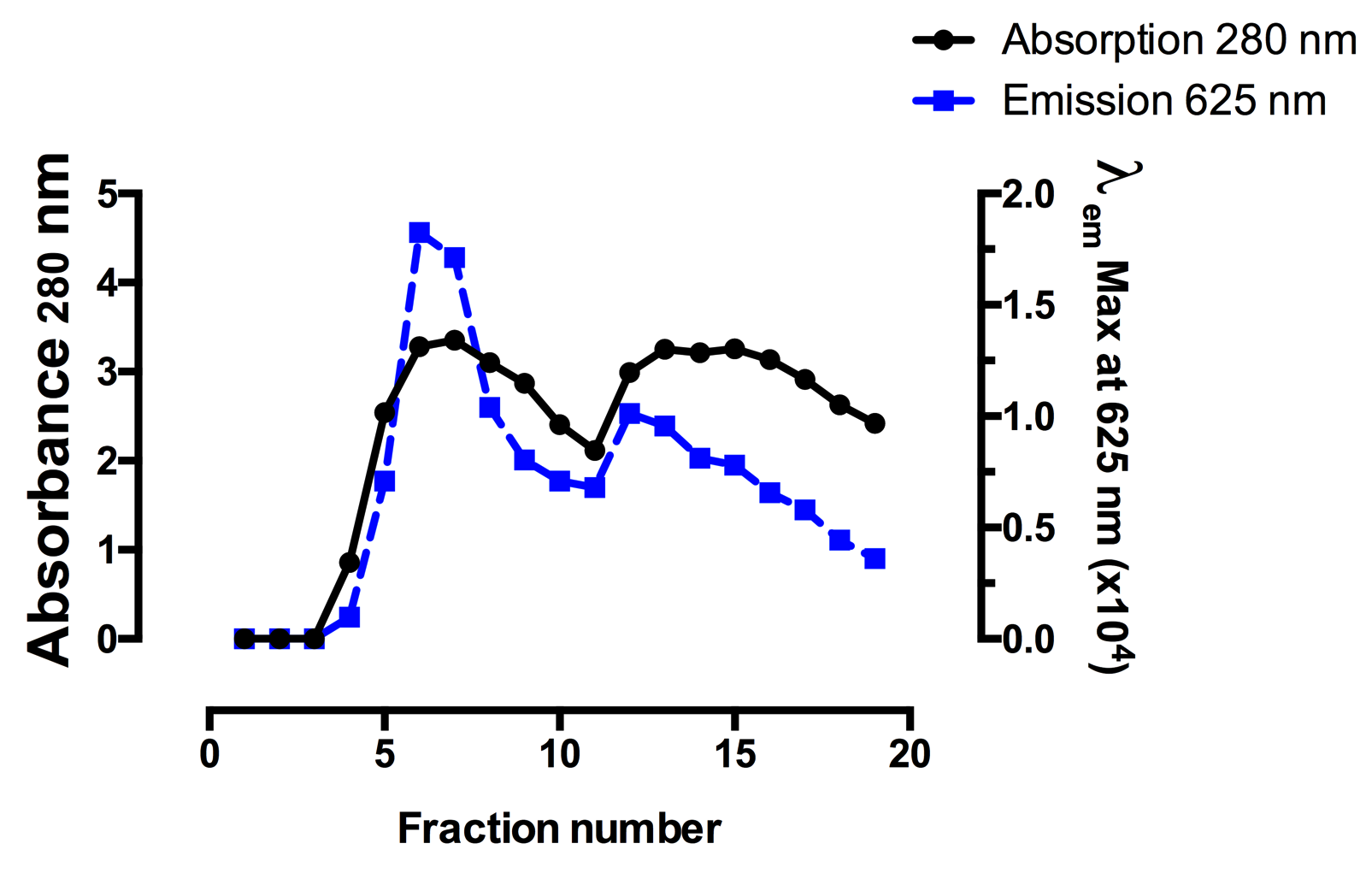

Supplement: S3 Fig — (TIFF) [file pone.0182926.s003.tiff]

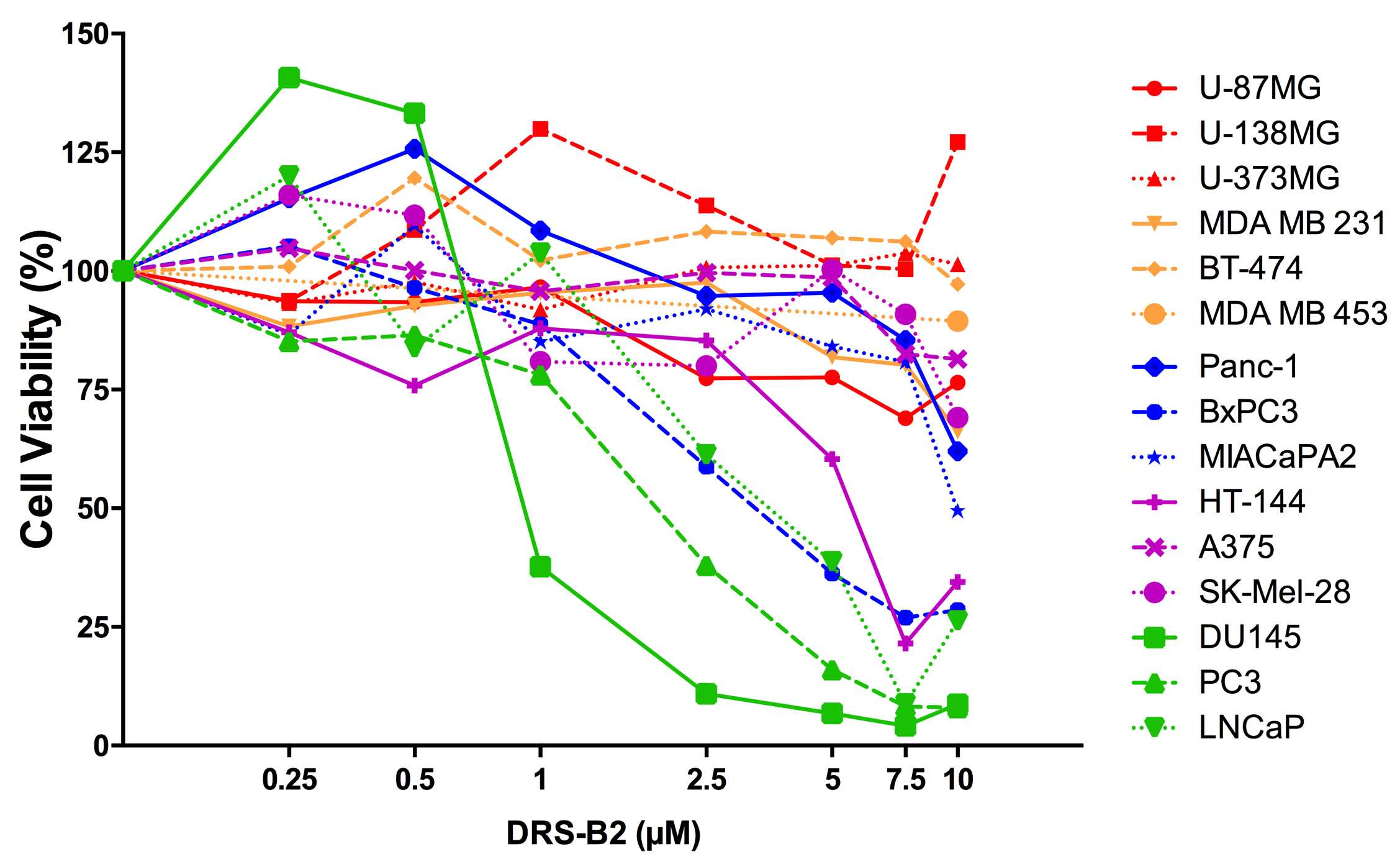

Supplement: S4 Fig — (TIFF) [file pone.0182926.s004.tiff]
